# Translocations contribute to population rescue in an imperiled woodpecker

**DOI:** 10.1073/pnas.2410946122

**Published:** 2025-07-28

**Authors:** Alexander L. Lewanski, Tyler Linderoth, Greg Thompson, Angela Tringali, Emily Angell, Reed Bowman, Sarah W. Fitzpatrick

**Affiliations:** ^a^W.K. Kellogg Biological Station, Michigan State University, Hickory Corners, MI 49060; ^b^Department of Integrative Biology, Michigan State University, East Lansing, MI 48824; ^c^Ecology, Evolution, and Behavior Program, Michigan State University, East Lansing, MI 48824; ^d^Archbold Biological Station, Venus, FL 33960

**Keywords:** translocations, pedigrees, demographic rescue, conservation

## Abstract

Anthropogenic destruction and fragmentation of habitat restrict many species to small, isolated populations, which often experience high extirpation risk. Restoring connectivity through translocations is one approach for mitigating the demographic and genetic perils faced by small populations. However, translocation interventions often lack substantial postrelease monitoring, and thus important information including the performance of translocated individuals, the long-term impacts on the recipient population, and the extent to which management objectives are fulfilled over time are often poorly known. Here, we examined the establishment dynamics and long-term outcomes of translocations from multiple donor populations into an intensively monitored population of the federally threatened red-cockaded woodpecker. We found evidence that translocations contributed to population growth and led to genetic admixture within the population. The translocated birds provided direct demographic benefits through high rates of establishment, breeding, and survival. We found that the survival and lifetime reproductive success of individuals were positively related to their amount of translocation ancestry, indicating that demographic benefits extended beyond the direct performances of the translocated birds. The translocations diversified the population’s genetic composition with the ancestry of most individuals in the latter years of the study deriving from multiple translocation donor populations. We found marked heterogeneity in the genetic contributions of translocated individuals and cohorts, leading to disproportionate representation of certain lineages. Encouragingly, despite some accumulation of inbreeding during the study, the translocations thus far have not substantially contributed to inbreeding. Our findings illustrate in precise detail how translocations can be an effective approach for managing imperiled taxa.

## Significance

Translocations—the human-mediated movement of individuals—have been used to counteract the genetic and demographic perils faced by small, isolated populations. However, posttranslocation population monitoring is often inadequate to thoroughly understand the outcomes of these management actions. In a long-term study of red-cockaded woodpeckers, we found that translocations yielded substantial and positive effects. Birds with translocation ancestry exhibited high survival and lifetime reproductive success, which likely played an important role in the population growth that occurred during and after translocations. The translocations also led to a highly admixed population involving ancestries from multiple translocation donor populations while not substantially contributing to inbreeding. These results demonstrate how translocation can represent an effective strategy for the management of imperiled taxa.

There is a sizable class of species for which persistence hinges on active human intervention (1). For example, an estimated 84% of species listed under the United States Endangered Species Act are considered conservation reliant (2). Various factors can lead to conservation reliance, such as habitat loss, overexploitation, and pollution. The shared feature among conservation-reliant species is that, were management to end, unmitigated threats would likely jeopardize their persistence. Conservation-reliant species pose challenges for contemporary conservation infrastructure because existing policies and funding are ill-equipped to address their prolonged needs (2, 3). An increasingly critical charge of conservation science is to develop approaches to efficiently and effectively manage species that require indefinite assistance.

One of the leading threats to species is the destruction, fragmentation, and degradation of habitat (4–6). For species that rely on the affected habitats, these changes can splinter their distributions into small, isolated populations. The remnant populations are frequently prone to extirpation due to a constellation of threats, including enhanced vulnerability to demographic and environmental stochasticity (7, 8), limited adaptive potential (9), elevated inbreeding and inbreeding depression (10), and accumulating genetic load (11).

Restoring connectivity is a promising approach for improving population persistence in fragmented landscapes. Migration and gene flow can directly counteract the demographic and genetic challenges that often accompany small population size (12, 13). Connectivity can also increase the frequency of habitat patch (re)colonization, which can enhance patch occupancy and persistence of the broader metapopulation (14–16). In landscapes transformed by human activity, opportunity to restore natural dispersal (e.g., via habitat corridors) is often limited, leaving translocations as a more realistic option to restore connectivity and gene flow (17).

Translocations are widely employed in conservation settings (18–20), and their value has been endorsed by natural resource management agencies and global conservation authorities (e.g., refs. 17 and 21). Nonetheless, fundamental knowledge gaps continue to limit the utility and application of translocations for the management of imperiled taxa (e.g., ref. 20). The potential benefits of conservation translocations—promoting a larger and more genetically diverse population, thereby reducing inbreeding and ensuing deleterious effects—are often well articulated. However, the extent to which these benefits are realized, how the impacts of translocations change over time, and the demographic and genetic mechanisms by which various outcomes arise are typically less understood.

A major contributor to the knowledge gaps is the limited nature in which translocation interventions are often monitored and evaluated. Most conservation translocations reported in the literature are based on monitoring that only extends for a short period of time (e.g., a handful of years) after the translocation release events (22, 23). Additionally, monitoring frequently focuses on narrow aspects of translocated individuals’ performances such as how fitness proxies (e.g., growth and survival) compare with residents of the recipient population (e.g., refs. 24 and 25). Critically, the effects of translocations on the population depend not only on the translocated individuals themselves but also their descendants, and a variety of plausible scenarios exist for how descendants of translocated individuals may perform. For instance, hybrid offspring of translocated and resident individuals may show elevated fitness relative to the resident population (e.g., due to heterosis and/or masking of deleterious recessive variants), but over multiple generations, the fitness of the individuals with ancestry from the translocation donor population could decline below the resident population and their translocated ancestors due to outbreeding depression. Thus, initial fitness benefits may not necessarily predict longer-term outcomes, and understanding ultimate effects of translocations is impossible when monitoring is brief, primarily focused on the translocated individuals, and not conducted with adequate frequency to accurately quantify fitness. Long-term and continuous study of the recipient population is infeasible for many translocation interventions (26). However, in the rare cases when it is possible, these efforts can provide a uniquely detailed view into the consequences of translocations and their multigenerational impacts on the population. Evaluation of translocation interventions based on long-term studies will help achieve a more complete understanding of how translocations alter populations, further refine the implementation of translocations in conservation settings, and effectively tailor these actions to different management objectives and attributes of the focal population.

In the current study, we investigate a small, isolated population of red-cockaded woodpeckers (*Dryobates borealis*) at Avon Park Air Force Range (hereafter *Avon Park*) in Florida, United States (*SI Appendix*, Fig. S1). The red-cockaded woodpecker is an obligate resident of mature, open pine woodlands in the southeast United States (*SI Appendix*, Fig. S2). Due to widespread habitat loss and degradation from human activities (e.g., deforestation, fire suppression), it severely declined following European colonization of the region and is now found in a limited number of disjunct populations (*SI Appendix*, Fig. S1). The red-cockaded woodpecker was among the first species to receive protections from the Endangered Species Act upon its ratification in 1973, which prompted a concerted range-wide recovery effort focused on habitat and population management, including interpopulation translocations (27). These efforts have led to the stabilization or growth of many populations (28), which motivated the recent downlisting of the species from endangered to threatened (29). Nonetheless, the viability of many populations depends on continued intervention and management (28). The red-cockaded woodpecker therefore remains a conservation-reliant species for which the study of translocations and their demographic and genetic consequences is especially pertinent.

Mirroring the trends in many other red-cockaded woodpecker populations, the Avon Park population dwindled to a perilously small size during the mid-to-late twentieth century. In 1997, managers began enhancing habitat at Avon Park, and in 1998, started regularly translocating birds into the population. From 1994 through the present, the population has been intensively monitored with annual censuses and nearly complete documentation of nesting outcomes and parent–offspring relationships. The duration, granularity, and comprehensiveness of this monitoring provides an outstanding opportunity to study the impacts of translocations in an imperiled species on a highly fragmented landscape.

Here, we investigate the long-term outcomes of translocations in the red-cockaded woodpecker population at Avon Park. We document the extent to which translocated birds successfully established and bred—an essential but precarious initial stage of translocation interventions (23). Next, we examine variation in fitness components, including whether survival and reproductive success differed between translocated and resident individuals and whether they varied based on the amount of ancestry from the translocation donor populations (hereafter *translocation ancestry*). These relationships reveal the extent and mechanisms by which translocations boosted individual vital rates and, in turn, promoted population growth. Last, we leverage the near-complete population pedigree and census information to examine the effects of translocations on the population’s genetic composition including how genetic contributions vary across translocated individuals and translocation cohorts and whether translocations have directly exacerbated inbreeding. Jointly addressing these objectives provides a unique, high-resolution appraisal of the effectiveness of translocations for endangered species recovery and management.

## Results

### Demographic Performance of Translocated Birds.

**Fig. 1.**
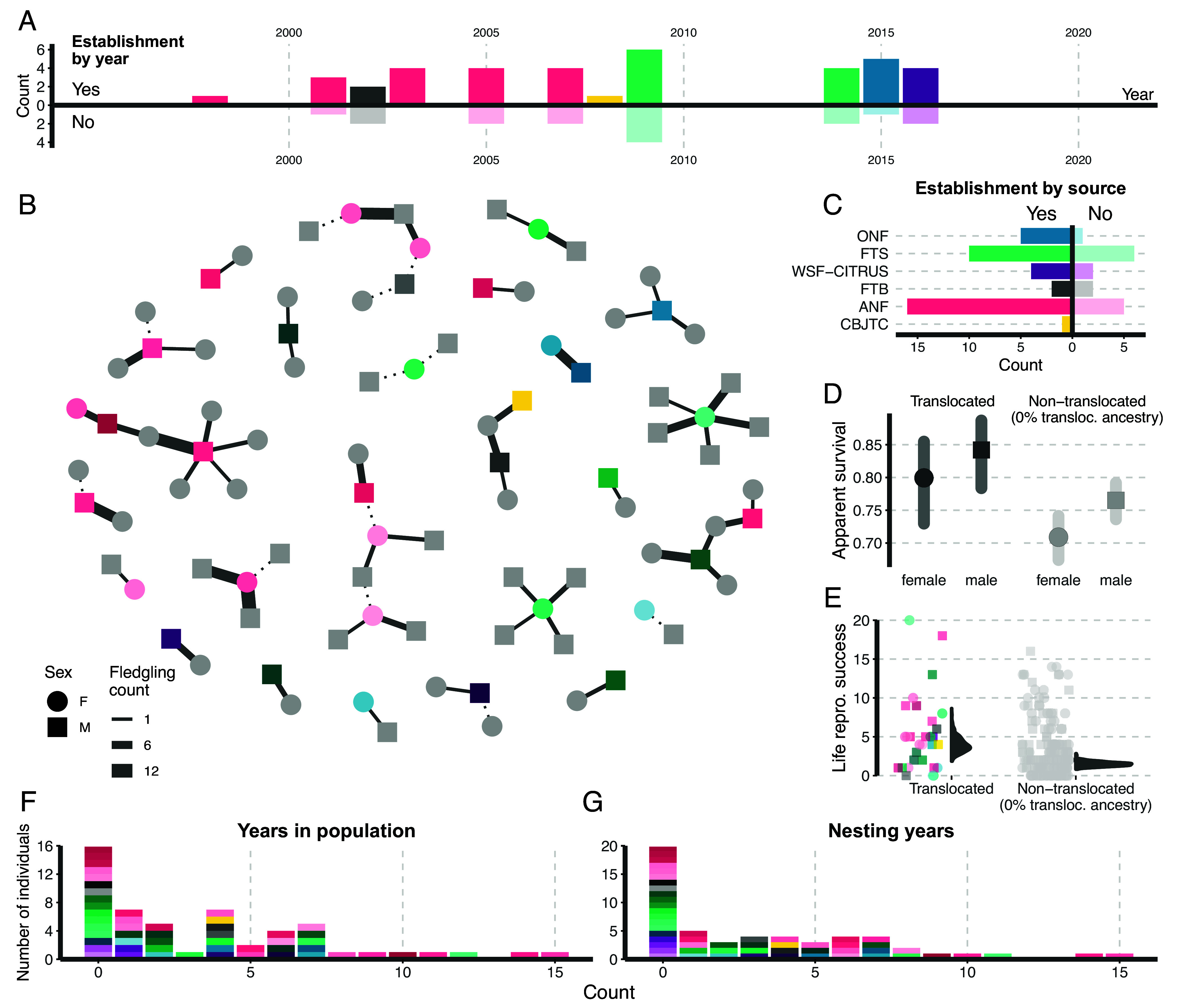
Establishment and reproductive activities of translocated birds. (*A*) Establishment outcomes for translocated birds organized by translocation year. The horizontal axis is year, and the vertical axis is diverging with counts of successfully established birds (i.e., recorded in at least one population census) above the horizontal axis and the birds that failed to establish shown below. (*B*) Network visualization of breeding pairs involving a translocated bird. Nodes represent individuals and are colored by source population (nontranslocated mates are shown in light gray). Edges indicate breeding pairs with thickness scaling with the number of fledglings produced across all nesting events involving the breeding pair. Dotted edges indicate breeding pairs that failed to produce fledglings. (*C*) Establishment outcomes of translocated birds organized by source population. The horizontal axis is diverging with the count of successful establishments shown on the left side of the vertical axis and failed establishments on the right. (*D*) Estimates with 95% CI of apparent survival for males and females that are translocated and nontranslocated with no translocation ancestry. (*E*) Lifetime reproductive success (total fledged offspring) of translocated vs. nontranslocated birds with no translocation ancestry. The points are the observed values, and gray distributions represent 500 expected values drawn from the posterior predictive distribution of a model examining differences in lifetime reproductive success between the translocated and nontranslocated birds. (*F*) Stacked barplot showing the total years that translocated birds were in the population. Each bird is represented by a bar and the color scheme matches panel *B*. Panel *G* is identical to panel *F* except that it shows the number of years that each translocated bird nested.

Between 1998 and 2016, 54 red-cockaded woodpeckers from six donor populations in a total of 11 translocation events were released into Avon Park (Fig. 1 and *SI Appendix*, Table S1). Translocation cohorts (i.e., individuals released at a single translocation event) ranged in size from 1 to 10. Overall, 38 individuals established (i.e., detected in at least one postbreeding census) and persisted in the population for 1 to 15 y (median: 4). Establishment percentages were generally high across donor populations (50 to 100%) and translocation events (50 to 100%). We found no statistically supported differences in establishment between sexes (Fisher’s exact test, *P* = 0.38), translocation events (Fisher’s exact test, *P* = 0.96), or donor populations (Fisher’s exact test, *P* = 0.81). Of the established individuals, most (n = 34; 89.5%) nested, ranging from 1 to 15 nesting years (median: 5). The translocated birds collectively produced 142 offspring that were detected in a census (and thus were recorded as part of the population) with per capita contributions for the established birds ranging from 0 to 17 (median: 3). Most breeding pairs with a translocated individual involved a locally hatched mate (translocated–local: 61 vs. translocated–translocated: 4), and most fledglings produced by translocated individuals were from translocated–local pairings (translocated–local: 170 vs. translocated–translocated: 13; Fig. 1*B*), which is desirable from the standpoint of generating admixture. Translocated birds had nearly 10 percent higher annual survival rates than birds without translocation ancestry based on capture–mark–recapture (CMR) modeling (Fig. 1*D* and *SI Appendix*, Table S11), and translocated breeders had higher lifetime reproductive success compared to locally hatched breeders without any translocation ancestry (mean parameter estimate = 0.984 [95% credible interval = 0.407, 1.586]; Fig. 1*E* and *SI Appendix*, Table S3).

### Population Expansion at Avon Park.

**Fig. 2.**
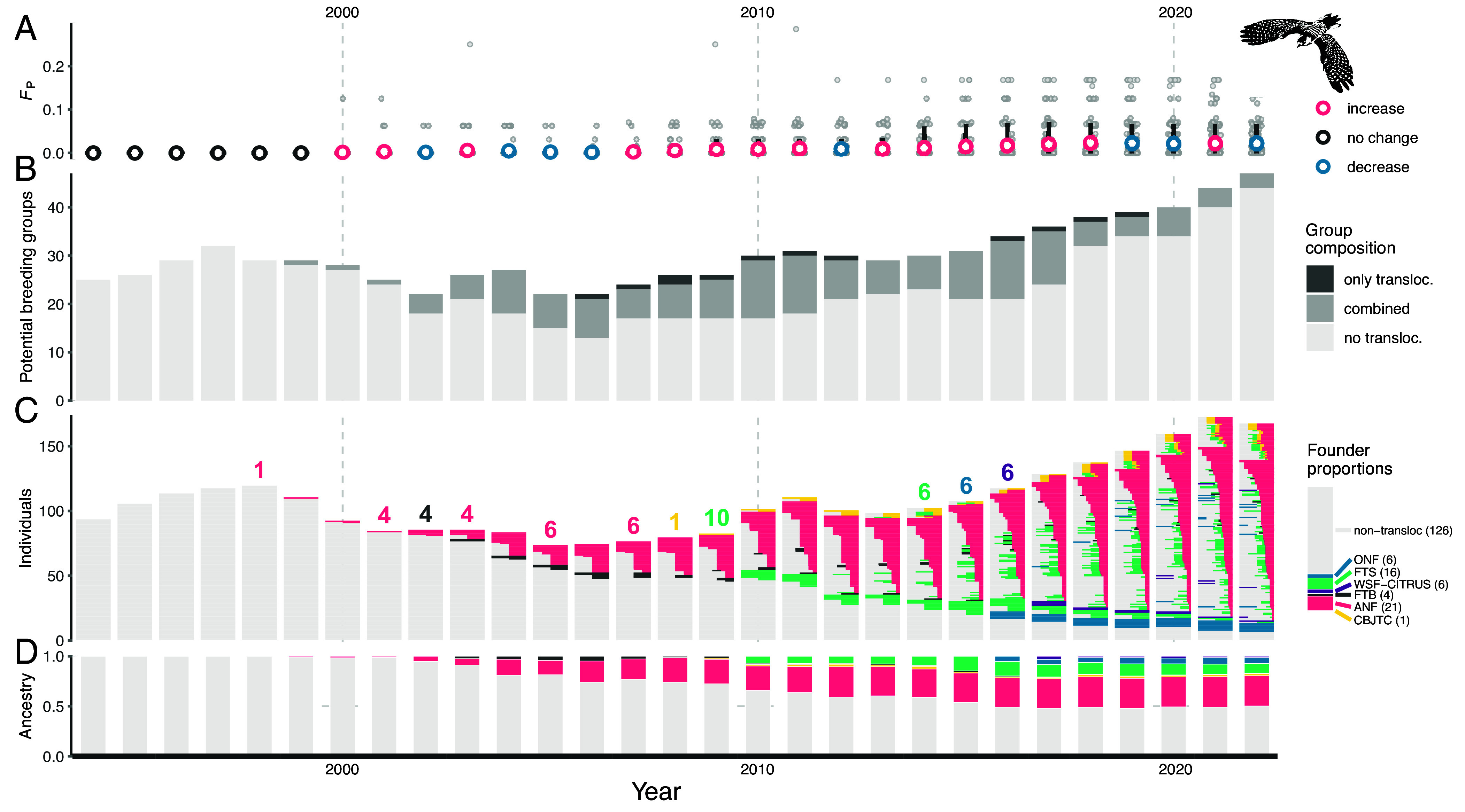
Summary of pedigree inbreeding (*F_P_*), expected ancestry, and number of individuals and potential breeding groups in the population. All plots involve a shared horizontal axis representing year. (*A*) Plot of expected inbreeding values of each year’s population. The light gray points are the inbreeding values of individuals. The larger, hollow points are the mean inbreeding values and are colored based on whether the mean inbreeding showed no change (dark gray), increased (magenta), or decreased (blue) from the previous year’s mean. The intervals represent the 10th and 90th percentiles of inbreeding values. (*B*) Barplots showing the number of potential breeding groups each year and whether they are composed of only translocated birds (*only transloc.*), only nontranslocated birds (*no transloc.*), or a combination of both (*combined*). (*C*) Barplots showing the proportion of ancestry that individuals are expected to inherit from each pedigree founder group (i.e., each translocation donor population and the nontranslocated founders). Each individual is represented as a thin, horizontal bar. The plot also indicates the total size of the population each year. The numbers above the bars indicate the year, cohort size, and donor population of translocation events. The legend to the plot’s right specifies the color corresponding to each pedigree founder group and also the proportion of founders in each group. The number next to each name indicates the count of individuals in that group. (*D*) Barplots showing the expected proportions of ancestry in the population (summarized across all individuals) from each pedigree founder group.

Both total population size and number of potential breeding groups grew over the monitoring period (Fig. 2). Potential breeding groups are a central focus in the management of red-cockaded woodpeckers because the effective size of a red-cockaded woodpecker population is more closely tied to the number of potential breeding groups than its census size (27). The population reached its lowest size of 73 individuals in 2005. Coincident with the bulk of translocations, the population then steadily grew (*SI Appendix*, Fig. S7) until it reached its peak size of 172 in 2021. The number of potential breeding groups ranged from 22 in the 2000s to 47 in 2022 (Fig. 2*B*). The number of potential breeding groups and population size showed similar changes through time (Spearman’s *r* = 0.90; *P* = 1.93e-11).

### Predictors of Survival and Reproductive Success.

**Fig. 3.**
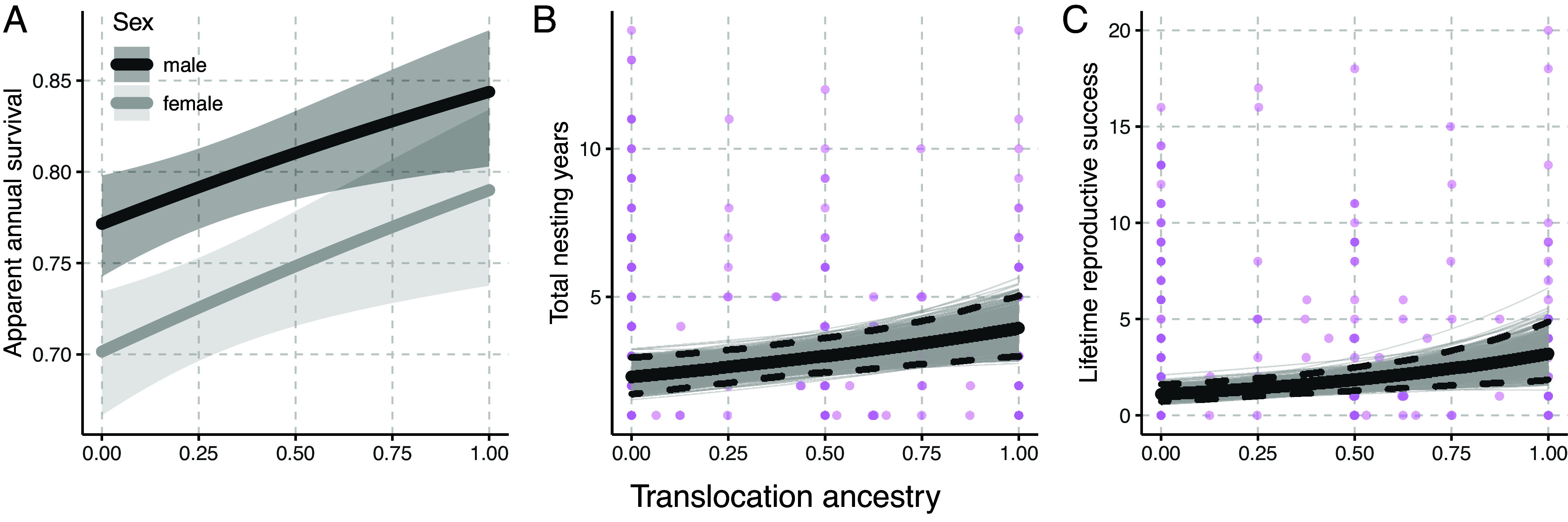
Estimated relationships between the proportion of ancestry that a locally hatched individual is expected to inherit from translocated individuals (translocation ancestry) and a series of fitness measures: apparent annual survival (*A*), total nesting years (*B*), and lifetime reproductive success (*C*). Each plot shows translocation ancestry along the horizontal axis and the fitness metric along the vertical axis. (*A*) The estimated apparent survival for each sex is shown as a line with the lighter bands representing the 95% CI. (*B* and *C*) In each plot, the bold, solid line shows the mean predicted relationship and the dashed lines represent the 95% credible interval. The lighter lines represent the predicted relationships based on 500 draws from the model’s posterior distribution. The models in panels *B* and *C* were based on locally hatched breeders and are plotted alongside the raw data (purple points).

We found that translocation ancestry was positively associated with several fitness metrics. Specifically, the best supported CMR model, which accounts for detection probability, included both sex and translocation ancestry effects. The model revealed higher annual survival for males compared to females [an established pattern in this species (27)], and survival tended to increase with greater translocation ancestry (Fig. 3*A* and *SI Appendix*, Table S13).

The Bayesian regression models of reproductive performance measures provided support for positive effects of translocation ancestry on both nesting years (mean parameter estimate: 0.541 [95% CI: 0.206, 0.888]; Fig. 3*B*) and lifetime reproductive success (mean parameter estimate: 1.049 [0.479, 1.643]; Fig. 3*C*) but no support for an effect of translocation ancestry on mean annual reproductive success. These relationships, paired with the finding that the lifetime reproductive success of individuals is significantly explained by the number of years that they nest (mean parameter estimate: 0.191 [0.173, 0.208]; *SI Appendix*, Fig. S10 and Table S15), suggest that birds with greater translocation ancestry tended to nest for more years and ultimately achieve greater reproductive success. The models also yielded well supported relationships involving other variables including a positive effect of group size on all three reproductive performance measures and a negative relationship with first calendar year of breeding and both lifetime reproductive success and total nesting years. We found no significant effect of the number of ancestral groups comprising an individual’s ancestry (a simple measure of admixture) on any of the reproductive measures (*SI Appendix*, Tables S8 and S9). In a supplementary analysis, we found a negative effect of probability of homozygous Avon Park ancestry on lifetime reproductive success (mean parameter estimate: −0.830 [−1.327, −0.352]; *SI Appendix*, Fig. S9 and Table S14). Additionally, based on the model that included only individuals with complete grandparent information, we did not find support for an effect of inbreeding (*F_P_*) on lifetime reproductive success (mean parameter estimate: 2.464 [−5.094, 9.841]; *SI Appendix*, Table S16).

### Ancestry Composition and Genetic Contributions of Translocations.

The establishment and reproductive activities of translocated birds substantially altered the ancestry of the population. Translocation ancestry rapidly increased during and after the 19 y period of translocations so that by 2022, nearly all living individuals (161/167) were expected to possess some translocation ancestry, and 49.8% of the population’s total ancestry could be traced back to donor populations (Fig. 2*D*). Ancestries from all donor populations were represented through 2022. However, translocation ancestry in the 2022 population was dominated by the ANF donor population (61.2%) and to a lesser extent the FTS population (20.4%), indicating uneven contributions across donor populations to the contemporary population.

The spread of translocation ancestry coincided with substantial mixing of ancestries within individuals (Fig. 2*C*). By 2022, the median number of pedigree founder groups (i.e., the donor populations and nontranslocated pedigree founders) from which an individual descended was three (*SI Appendix*, Fig. S8*B*). Correspondingly, starting in 1999 (after the first translocation), *m_d_* (a metric of ancestry mixing) steadily grew at a mean ± SD rate of 0.03 ± 0.064 per year, peaking at 0.688 in 2022 (*SI Appendix*, Fig. S8*A*).

**Fig. 4.**
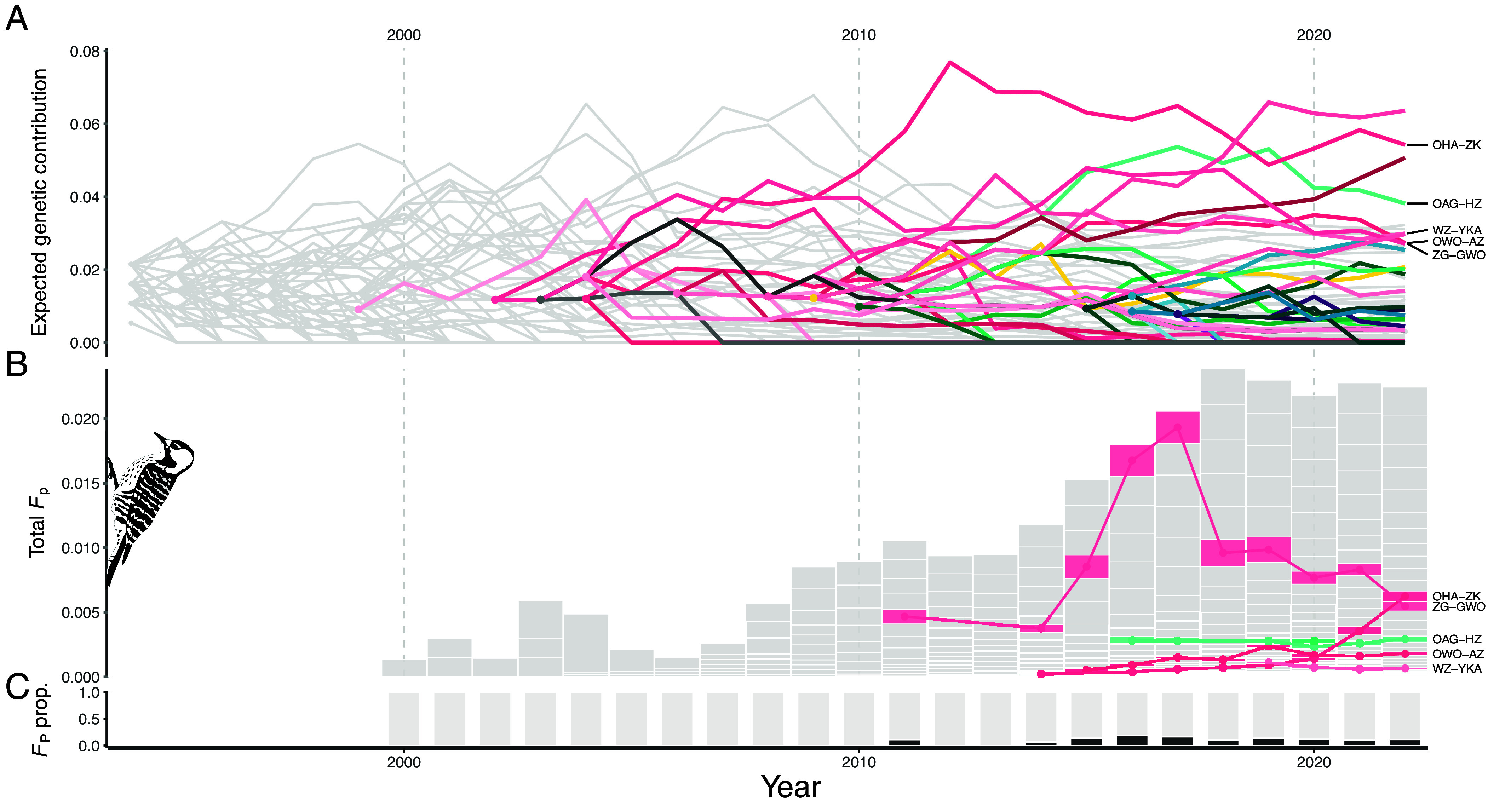
The genetic and inbreeding contributions of pedigree founders to the population. All plots involve a shared horizontal axis representing year. (*A*) and (*B*) share a color scheme with translocated individuals uniquely colored based on source population and shade. All nontranslocated founders are shown in light gray. (*A*) The expected genetic contribution of each pedigree founder in the population. Each individual’s contributions across years are connected by a line. (*B*) Stacked barplots showing, across all individuals in a year’s population, the total amount of pedigree inbreeding that can be attributed to each pedigree founder (each bar in the stack represents the contribution of an individual). When a translocated bird contributes to inbreeding across >1 y, its contributions are connected by a line. The inbreeding values are scaled by the number of individuals in the population. The contribution value for a particular pedigree founder can thus be interpreted as the probability that the two alleles at a randomly chosen loci in a randomly chosen individual are identical by descent due to alleles inherited from the founder. (*C*) A summary of panel *B* showing the proportion of pedigree inbreeding that can be attributed to translocated birds (dark gray) and nontranslocated pedigree founders (light gray). Panels *A* and *B* include labels for the five translocated birds that contribute to inbreeding in the final monitoring year.

**Fig. 5.**
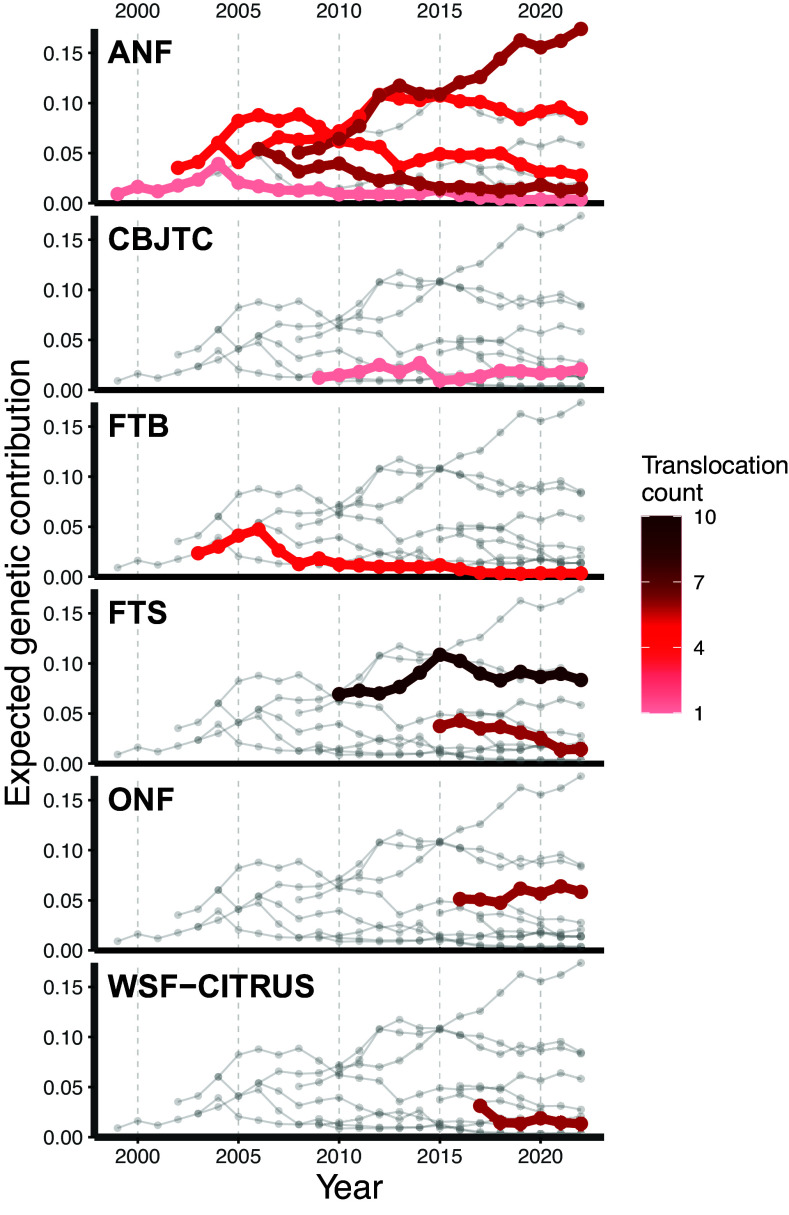
Expected genetic contributions of translocation cohorts (vertical axis) to the population through time (horizontal axis). Each series of connected points represents the contributions of a single cohort (i.e., the set of individuals translocated in a single year). A plot is included for each of the six donor populations. In each donor population’s plot, the contributions of cohorts sourced from the corresponding population are colored based on the number of individuals included in the cohort (darker red corresponds to more individuals). The contributions of cohorts originating from a nonfocal donor population are shown in light gray.

We found high variation in expected genetic contributions of individuals and translocation cohorts (the bird(s) released at a single translocation event) through time (Figs. 4*A* and 5). Many of the translocated birds that established (n = 14) were expected to provide no genetic contributions by the final monitoring year. Although these dead-ends are inevitable for deceased birds that never nested, the noncontributors included 10 individuals that nested and 6 individuals with offspring that were recorded in a population census. Conversely, several translocated birds and cohorts displayed notably large contributions. For example, individual OHA-ZK was among the top three contributors in every year from 2010 to 2022, and in seven of the eight final monitoring years (2015 to 2022), the top five contributors were translocated birds. There was also substantial heterogeneity among the trajectories of translocation cohorts. All cohorts were still represented in the 2022 population. However, the contributions of multiple cohorts consistently waned through time (e.g., 2003 FTB, 1998 ANF, and 2015 FTS cohorts) while several showed fairly stable contributions (e.g., 2009 CBJTC and 2010 FTS cohorts). A notable outlier was the 2008 ANF cohort, which displayed large contributions that consistently grew through 2022.

### Inbreeding.

Pedigree inbreeding (*F_P_*) slowly accumulated over the monitoring period (mean annual increase in average *F_P_* of 0.001 ± 0.002 SD since 2000; Fig. 2*A*). The increase in average *F_P_* was accompanied by a growing percentage of individuals with nonzero *F_P_* values (increasing at a mean ± SD rate of 2.48 ± 2.97% per year starting in 2000). We additionally identified the pedigree founders to which *F_P_* can be traced back and quantified the proportion of *F_P_* that can be attributed to each of these ancestors (Fig. 4 *B* and *C*). From 2011 (the first year a translocated bird contributed to *F_P_*) to 2022, the percentage of *F_P_* that could be traced back to translocated birds ranged from 0% (2012 and 2013) to 18.6% in 2016. Throughout the monitoring period, only six translocated birds ever contributed to *F_P_*, and these corresponded to some of the largest genetic contributors (Fig. 4). However, the translocated birds did not generally represent the largest contributors to *F_P_*, with only one individual ranking in the top five contributors to *F_P_* in a given year and only two ranking in the top 10. These results indicate that, despite the high reproductive success of multiple translocated birds and their offspring, the translocations have not substantially elevated inbreeding.

## Discussion

Here, we leveraged a long-term demographic study of a red-cockaded woodpecker population to understand the outcomes and impacts of conservation translocations. We demonstrate that translocations fulfilled their objectives of demographic and genetic augmentation while largely avoiding unwanted repercussions. Further, empowered by the comprehensive and extended nature of the monitoring, we found marked discrepancies in how different translocation events and translocated individuals contributed to the population through time. Our study illustrates the substantial and complex ways in which translocations can influence the demography and genetic makeup of a population, and it adds to the modest but accumulating collection of case studies documenting the extended utility of translocations in real-world management efforts (e.g., refs. 30–32).

### Translocations Contribute to Demographic Rescue.

We found considerable evidence that translocations contributed to demographic rescue of the population at Avon Park. First, the translocations boosted the population directly through the establishment and overall high survival and reproductive performances of the translocated birds, which surpassed the individuals at Avon Park with no translocation ancestry. Notably, the demographic benefits of translocations extended beyond the direct impacts of the translocated birds. Locally hatched birds with more translocation ancestry tended to survive better, and lifetime reproductive success tended to increase with greater translocation ancestry among locally hatched breeders. Critically, the positive demographic impacts of the translocations corresponded to increases in the population census size and number of potential breeding groups (Fig. 2 *B* and *C*). The relationship between translocation ancestry and lifetime reproductive success seemed to emerge via birds nesting for more years and consequently fledging more offspring over their lifetimes, rather than producing more fledglings in each year that they bred. These results provide clear evidence that translocation ancestry was associated with several important components of fitness and sets the stage for future work to more comprehensively examine the fitness impacts of translocation ancestry in this population, such as dissecting survival at different ages and examining how translocation ancestry relates to transitions between life stages (e.g., nonbreeder to breeder).

Several potential explanations exist for the elevated fitness of translocated birds and their descendants. One possibility is that the genetic material from translocated birds masked the expression of recessive deleterious mutations that had drifted to high frequency while the Avon Park population was small and declining prior to translocations (11, 33). The translocated birds were likely less inbred as they were intentionally sourced from larger populations with potentially less drift load. In accordance with this explanation, we confirmed that an individual’s probability of homozygous Avon Park ancestry showed a negative relationship with lifetime reproductive success (*SI Appendix*, Fig. S9*B*), consistent with a realized Avon Park genetic load, while the probability of homozygous Avon Park ancestry was negatively correlated with translocation ancestry (*SI Appendix*, Fig. S9*A*). This explanation aligns with how inbreeding depression is thought to chiefly arise (34) and is consistent with the primary way in which gene flow into small populations is expected to elevate fitness over the short term (i.e., by alleviating inbreeding depression) (35).

Notably, we failed to find evidence for a relationship between pedigree inbreeding and lifetime reproductive success, though this does not invalidate the potential relief of inbreeding depression discussed above. For instance, it could be difficult to detect inbreeding depression based on inbreeding coefficients if deleterious recessive mutations were at high frequency, and thus most individuals were homozygous for the mutations regardless of their inbreeding value. In this case, only breeding between individuals from different populations would reveal the existence of inbreeding depression (36). In fact, existing evidence of fitness declines with increasing pedigree inbreeding in red-cockaded woodpeckers primarily derives from a substantially larger population than Avon Park (37, 38), and thus the impacts of drift and the prevalence of deleterious genetic variation likely differ between the populations. Another possible explanation for the apparent lack of fitness relationships with inbreeding is that, because we can only recover the modest amount of inbreeding that has arisen in the pedigree documented during monitoring, we are missing a substantial amount of relevant inbreeding variation that stems from deeper, unrecorded portions of the population’s pedigree.

There are other potential reasons for the improved fitness of individuals with translocation ancestry, which could operate concurrently with the masking of deleterious mutations. Overdominance could contribute to the stronger performance of individuals with translocation ancestry because these individuals were often admixed with ancestries from Avon Park and one or more translocation donor populations (34). It is also possible that the translocated birds (re)introduced advantageous mutations that were absent at Avon Park when monitoring started, and therefore those with translocation ancestry benefited from these mutations. Genomic investigations of the Avon Park and translocation donor populations will help further decipher the genetic mechanisms underlying fitness effects of translocations.

### Translocations Diversify the Population’s Genetic Composition.

A potential benefit of translocations is an increase in the genetic diversity of the recipient population, without swamping the population with few translocated lineages. We found strong evidence that this outcome was achieved for red-cockaded woodpeckers at Avon Park. The translocations substantially altered the population’s genetic composition, and these genetic impacts persisted throughout the monitoring period. The translocation ancestry grew to represent ∼50% of the population’s expected genetic ancestry by the final monitoring year and involved substantial mixing of ancestries from multiple donor populations within individuals. Although we did not find support for a positive effect of an increasing number of ancestry groups (i.e., individual admixture) on fitness, the admixture we observed may still offer benefits such as increasing the population’s adaptive potential over longer timescales (39).

Detailed tracking of the genetic contributions of translocated individuals and cohorts helped distinguish between scenarios by which translocation ancestry was maintained in the population. For instance, because the translocations occurred across multiple events spanning about two decades, it is plausible that translocation ancestry was maintained by the regular input of individuals even if their contributions to the recipient population were largely transient (e.g., the translocated birds and their descendants had little reproductive success and thus their ancestry was rapidly lost). In contrast, we found that many translocated individuals across multiple cohorts and donor populations provided sustained contributions including several translocated individuals that emerged as the largest genetic contributors to the contemporary population across all pedigree founders. Appreciable legacies of many translocation events were therefore reflected in the population’s genetic composition through the latter monitoring years.

A potentially important consideration for variation in genetic contributions among individuals and cohorts is the substantial changes in population size and the timing of these changes relative to when translocation cohorts were introduced into the population. For example, a period of sustained growth following a translocation would likely promote the maintenance of the translocated birds’ ancestries compared to a scenario of population decline following a translocation event. In other words, extrinsic factors regulating population growth likely played some role in the success, or lack thereof, of translocated lineages. Nonetheless, despite the complex and stochastic set of factors that likely influenced the genetic contributions of translocated birds, we found that their genetic contributions to the population were predictable to some degree. For example, a strong, positive relationship exists between the lifetime reproductive success of translocated birds (i.e., a short- to medium-term fitness measure) and their genetic contributions to the population in the final monitoring year (i.e., their longer-term contribution) (*SI Appendix*, Fig. S11 and *Reproductive Success and Genetic Contributions*). This relationship indicates that translocated individuals that were more reproductively successful also tended to provide larger genetic contributions to the contemporary population.

### Translocations Minimally Increase Inbreeding.

Inbreeding is a persistent focus in small population management (10), and the alleviation of inbreeding and inbreeding depression is sometimes cited as a motivation to implement translocations. However, translocations into small populations can involve risk. If ancestry from a few migrants with disproportionate reproductive success predominates in the population, the translocations may reduce the population’s effective size (40) and exacerbate inbreeding. High reproductive skew is especially common in despotic cooperative breeders like red-cockaded woodpeckers as a byproduct of their breeding systems (41, 42). Moreover, migrant-driven inbreeding has been documented in other species with propensities for reproductive skew including in gray wolves (43) and recently in a Florida Scrub-Jay population formed via mitigation translocations (44), underscoring how this phenomenon is a reasonable concern in small populations.

We found that a small but appreciable amount of inbreeding emerged in the Avon Park population during the monitoring period. However, we failed to find strong evidence that the translocations consistently elevated inbreeding. A subset of translocated birds, representing some of the largest genetic contributors across all pedigree founders, eventually contributed to inbreeding. However, the largest contributors to inbreeding trace back to nontranslocated (i.e., local) founders (Fig. 4). These results indicate that, despite the disproportionate success of certain translocated lineages, translocated birds have so far not appreciably exacerbated inbreeding within the population.

### Lessons for Conservation Translocations in the Fragmented Anthropocene.

The translocations at Avon Park offer general lessons for the use and implementation of conservation translocations in red-cockaded woodpeckers and other species found in small, isolated populations. First, our findings highlight how the genetic impacts of translocations (and genetic composition more generally) can be dynamic in small populations. This volatility means that both desired (i.e., increased genetic variation, masking of deleterious alleles) or undesired (i.e., genomic sweeps, elevated inbreeding, or loss of translocated individuals’ genetic contributions) outcomes can arise quickly. Thus, regular monitoring of populations undergoing translocation interventions is critical for detecting and diagnosing emerging problems. Although it may be infeasible for the vast majority of translocation interventions to include monitoring as comprehensive as the efforts at Avon Park, lower intensity monitoring strategies, such as periodic demographic and genomic sampling, can be effective for detecting important changes in a population following translocations (44).

Translocations are often implemented as a crisis tactic for thwarting imminent extirpation—an initial impetus for the Avon Park translocations. However, as has been advocated by others (45, 46), translocations can provide more general value for restoring connectivity and maintaining long-term population viability. For many populations, even if not perilously small, regular gene flow will be necessary for preventing the problematic elevation of inbreeding and loss of genetic variation. As observed in the Avon Park red-cockaded woodpeckers, the slow growth of inbreeding during and after the translocations (including throughout a period of sustained population growth) demonstrates that a single translocation event (or small number of translocations) will likely fail to permanently alleviate inbreeding (47). For this species and the many others found in highly fragmented landscapes, regular pulses of assisted migration may ultimately be necessary to prevent undesirable genetic changes within populations.

The inbreeding results are especially pertinent for the range-wide management of red-cockaded woodpeckers. Currently, regulations governing the management of this species specify translocations should be limited to populations with fewer than 30 potential breeding groups (27). The finding of inbreeding accumulation in the Avon Park population, despite exceeding the potential breeding group threshold, suggests that inbreeding (and related fitness consequences) could pose a threat, even to populations that exceed the maximum size threshold for translocation eligibility. Thus, reevaluation of this guideline in the U.S. Fish and Wildlife Service Recovery Plan for red-cockaded woodpeckers may be warranted. More generally, translocations may be a valuable strategy for restoring connectivity and managing populations of various sizes (not only the perilously small).

## Conclusions

As human activities further fragment habitat and disrupt natural connectivity, it is vital for management to be effectively tailored to isolated populations. We presented evidence that carefully implemented translocations into the red-cockaded woodpecker population at Avon Park offered a swift demographic boost and provided extended genetic and demographic benefits via the translocated individuals’ descendants. Although translocations are an active (or planned) component of managing many imperiled taxa, their use has often not been explicitly motivated by the potential longer-term genetic and demographic benefits that translocations could confer (20). The findings of this project, which focused on a real-world conservation scenario within an iconic, imperiled species, provide further motivation for thoughtful implementation of translocations to intentionally target these potential long-term benefits. Importantly, the population improvements documented here reflect the combined effects of multiple management actions, including consistent efforts to enhance and expand habitat for red-cockaded woodpeckers at Avon Park. Pairing translocations with habitat management efforts to enable larger population sizes over extended time frames is crucial for maximizing the benefits derived from translocations. The ongoing implementation and evaluation of translocation interventions will help further hone the effectiveness of this approach for the management of imperiled taxa.

## Materials and Methods

### Study System Overview.

The red-cockaded woodpecker is an endemic bird species of the southeast United States where it is a specialist of mature, open pine woodlands (*SI Appendix*, Fig. S2). Red-cockaded woodpeckers live in cooperatively breeding groups composed of a breeding pair and up to four helpers that together occupy and defend a set of cavity trees called a *cluster* (48). The species was historically common with an estimated range-wide population of >900,000 groups at the start of European settlement in the region. However, habitat loss and deterioration stemming from human activities including fire suppression, development, and forestry triggered a severe decline. By the last quarter of the twentieth century, the range-wide population had dropped to ≤4,000 breeding groups comprising ≤10,000 individuals, which were mostly distributed as small, isolated populations (29, 49). Red-cockaded woodpeckers were listed as federally endangered in 1973, and ensuing management efforts successfully promoted the recovery of many populations. As of 2018, the range-wide population is estimated to include 10,000 to 30,000 individuals distributed across ≥8,000 clusters within 124 populations (29, 50).

Here, we focus on a population of red-cockaded woodpecker at Avon Park Air Force Range, a military installation in central Florida, United States. The Avon Park population was reduced to <30 potential breeding groups (a set of birds including at least one adult male and female occupying a cluster) in the early 1990s. Starting in 1998 and occurring regularly through 2016, subadults (<1 y old) from populations in Georgia and northern Florida were translocated into Avon Park in the fall for the purposes of genetic and demographic augmentation. Translocations either involved male–female pairs that were released into unoccupied, recruitment clusters (containing four artificial cavities) or single females with the goal of pairing them with single, nontranslocated males. The population has experienced other management actions spanning the time period of translocations including artificial cavity construction in occupied clusters and habitat maintenance and restoration (e.g., prescribed burning). As part of our analyses, we also included red-cockaded woodpeckers at River Ranch, a private tract of land abutting Avon Park. The River Ranch birds were included in the Avon Park monitoring efforts because they were contiguous and therefore part of the Avon Park population. However, River Ranch did not receive any habitat, cavity resource, or population management, and red-cockaded woodpeckers were extirpated from that area by 2013.

### Population Monitoring.

Beginning in 1994 and continuing through the present (this paper’s scope is 1994 to 2022), the Avon Park population has been intensively monitored. The vast majority of birds have been uniquely banded and thus are visually identifiable to the individual level. Nearly all nests were monitored until fledging or failure, which provides extensive information on reproductive behaviors and outcomes. We also use the nesting data to construct the population’s pedigree based on the identities of breeders and young at each nest. The accuracy of this pedigree (and of downstream analyses) presupposes that the observed breeders of a nest represent the nestlings’ true parents. This assumption is justified in red-cockaded woodpeckers because they are highly monogamous within breeding seasons and engage in minimal extrapair reproduction (51, 52). Following each breeding season, a census was conducted to determine the size and individual-level composition of the population. These censuses were nearly exhaustive with few individuals apparently being missed each year. We provide details on evaluation and processing of the census and pedigree data in *SI Appendix*, *Supplementary Background and Methods*.

### Population-Level Demographic Patterns.

For each year, we calculated the population’s census size and number of potential breeding groups to document population-level changes during the course of monitoring. We quantified census population size as the tally of all surviving adults and juveniles based on postbreeding censuses, and we calculated interyear population size change as the percentage difference in size between adjacent years. We quantified the degree of correspondence between population size and potential breeding group count using Spearman’s correlation.

### Direct Performance of Translocated Birds.

To characterize the direct performances and breeding activities of translocated birds following their release at Avon Park, we first examined the extent to which translocated birds successfully established by calculating the percentage of successful establishments across sexes, translocation years, and donor populations and the number of years that each bird existed in the population. We designated a bird as successfully established if it was recorded in a postbreeding census, which indicates that it persisted in the population through at least one breeding season. For each of these groupings, we tested for differences in establishment success using Fisher’s exact tests. To summarize the reproductive activities of translocated birds, we tallied the number of years that each individual nested. We then recorded the number of nests and nestlings produced by types of breeding pairs involving at least one translocated bird—pairs where both partners were translocated and pairs involving a translocated and nontranslocated bird.

We also conducted a series of analyses to compare the survival and reproductive performances of translocated birds to the resident population at Avon Park with no translocation ancestry. First, we fit CMR models with Program Mark (53) and RMark (54) to test for variation in survival between translocated individuals and Avon Park birds with no translocation ancestry, while accounting for imperfect detection. Second, we fit a series of Bayesian regression models with stan (55) and brms (56) to examine whether translocated birds showed differences in lifetime reproductive success (total fledglings produced over an individual’s life) compared to birds with no translocation ancestry (*SI Appendix*, Tables S2 and S3). We provide additional details in *SI Appendix*, *Supplementary Background and Methods*.

### Expected Inbreeding, Ancestry, and Genetic Contributions.

For all analyses of expected inbreeding, ancestry, and genetic contributions, we made the standard assumptions that all pedigree founders (i.e., individuals without parental information) were noninbred and lacked coancestry. The pedigree founders include the existing individuals in the population at the start of monitoring, a few likely natural migrants, and translocated birds. Hereafter, we refer to the six donor populations and the nontranslocated birds (original population constituents and natural migrants) as the *pedigree founder groups*. We use the familiar pedigree-based definition of *F_P_*, which can be defined as the proportion of the genome that is expected to be identical by descent (IBD) based on common ancestry within the observed pedigree.

#### Genetic composition.

We examined the ancestry of the population to explore the genetic impacts of translocations on the population. First, we used single locus gene drop simulations (57) to calculate the proportion of ancestry that each individual and each year’s population derives from translocated birds. For gene dropping, each pedigree founder was assigned two unique alleles (a paternal copy and maternal copy). The mendelian transmission of alleles was then simulated through the pedigree (for each individual, choosing at random the allele copy that it inherits from each parent). The gene dropping process was repeated 50,000 times. For each individual, we calculated the extent of ancestry from each pedigree founder group as the proportion of alleles across gene drop runs that were inherited from each group. To quantify translocation ancestry, we calculated the proportion of alleles inherited from translocated birds.

We further characterized the translocations’ impacts on the genetic composition of the population by assessing the degree to which ancestry from the different translocations has been integrated at the individual level. First, as a simple summary measure, we counted the number of pedigree founder groups from which each individual was expected to inherit alleles based on the gene drop simulations. Second, we used *m_d_* (58) to quantify the degree to which ancestries from the different pedigree founder groups were evenly distributed across individuals. The *m_d_* metric ranges from zero to one with zero occurring when ancestries from different groups are completely unmixed within individuals (i.e., each individual’s ancestry is limited to one group) and one occurring when ancestries from different groups are evenly mixed among individuals. We calculated *m_d_* based on the proportion of alleles that individuals were expected to inherit from each pedigree founder group from the gene drop simulations. We first calculated *m_d_* for the 1999 population because this was the first year when translocation ancestry was present in the population, and *m_d_* is undefined when ancestry from only one group exists.

We quantified the genetic contributions of translocated birds using the approach introduced by ref. 59, which uses additive genetic relatedness matrices derived from the pedigree to estimate individuals’ genetic representations in the population. For each pedigree founder, we calculated its contribution to each year’s population starting with its first year in the population during the monitoring period. We calculated the contributions of translocation cohorts by summing the contributions of individuals comprising each cohort. We standardized contribution values based on the size of each year’s population (59).

#### Inbreeding.

We examined *F_P_* to: 1) document its extent and change over time; and 2) quantify the degree to which translocated birds have directly contributed to *F_P_*. We used the tabular method (60) to calculate *F_P_* of each individual from the pedigree. To quantify the contributions of translocated birds to inbreeding, we used the gene dropping simulations to ascribe the extent of *F_P_* that was contributed by each pedigree founder. For each gene drop iteration, we calculated a founder’s *F_P_* contribution to a particular year’s population by tallying the individuals that have IBD alleles (possessing two copies of the same allele inherited from the focal founder) and dividing this value by the population size. We calculated this quantity for each run and then calculated the average across simulations. Because the *F_P_* count was standardized by the count of individuals, this quantity can be interpreted as the probability that the two alleles at a randomly chosen locus in a randomly chosen individual are IBD due to the founder. For every pedigree founder, we completed these calculations for each year’s population.

### Predictors of Survival and Reproductive Success.

We conducted several analyses to assess the predictors of several fitness measures with the primary goal of establishing whether the translocations provided demographic and fitness benefits to the Avon Park population. We limited these analyses to birds hatched at Avon Park to focus on the effects of translocations beyond the immediate outcomes of the translocated birds. Additional modeling details are provided in *SI Appendix*, *Supplementary Background and Methods and Supplementary Results*.

To investigate survival of all locally hatched birds, we fit CMR models including the following predictors: year, sex, and translocation ancestry. The set of models examined included all additive and interacting effects of the predictors on survival and incorporated either constant or time-varying recapture probabilities. We compared model performance with AICc and interpreted parameter estimates from the top performing model.

Next, we examined the predictors of three reproductive performance measures: total number of nesting years, mean annual reproductive success, and lifetime reproductive success. We limited these analyses to breeding birds that hatched at Avon Park during the monitoring period. For each breeding performance measure, we fit a series of Bayesian regression models with each reproductive measure as the response. We considered six predictors (two related to translocations and four other potentially relevant variables): translocation ancestry, number of pedigree founder groups from which an individual is expected to inherit genetic material (ancestry count), mean group size across an individual’s nesting events (mean group size), sex, first calendar year of nesting, and *F_P_*. We fit models with different predictor combinations and then compared models using expected log pointwise predictive density (ELPD) estimated with leave-one-out cross-validation (*SI Appendix*, Tables S4–S7). We identified all predictors found across competitive models [ELPD difference < 4 (61)] and interpreted parameter estimates based on the model with all of these predictors. Last, we fit a model with lifetime reproductive success as the response and nesting years and mean annual reproductive success as predictors to establish whether these two measures of reproductive performance show strong relationships with lifetime reproductive success. This model strengthened our capacity to draw connections between the different reproductive measures and the relationships that emerged in each set of models. All Bayesian models were fit with the genetic additive relatedness matrix included as a random effect and breeders alive in the last monitoring year included as right-censored observations.

We completed two supporting analyses to aid in our interpretation of the reproductive performance models. First, we explored whether the estimated relationship between lifetime reproductive success and *F_P_* (or lack thereof) may be influenced by pedigree depth because the models described above included some individuals with insufficient pedigree depth to detect inbreeding (e.g., individuals that only have parental information). We thus refit the aforementioned model that examined the predictors of lifetime reproductive success (*SI Appendix*, Table S8) with the dataset reduced to individuals with complete grandparent information in the pedigree. Second, as explored in the Discussion section, a relationship between translocation ancestry and fitness could potentially reflect a masking of deleterious recessive variants at high frequency in the Avon Park population at the start of monitoring. To more strongly demonstrate this connection, we refit the lifetime reproductive success model with the translocation ancestry variable replaced with the probability that an individual is homozygous for Avon Park ancestry. For each individual, the probability of homozygous Avon Park ancestry was calculated as the proportion of gene drop simulations in which it inherited two alleles originating in nontranslocated pedigree founders.

## Supplementary Material

Appendix 01 (PDF)

## Data Availability

Data and code associated with the paper are deposited on Zenodo (https://doi.org/10.5281/zenodo.15198584) (62).
