# Supplementary material for: Translocations contribute to population rescue in an imperiled woodpecker
Source: Proc Natl Acad Sci U S A. 2025 Jul 28;122(31):e2410946122. doi: 10.1073/pnas.2410946122 (PMC12337267; doi:10.1073/pnas.2410946122)
Supplement: Supplementary file 1 — Appendix 01 (PDF) [file pnas.2410946122.sapp.pdf]

Supplementary information: Translocations contribute to population rescue in an imperiled woodpecker

Authors:

Alexander L. Lewanski[1,2,3], Tyler Linderoth[1], Greg Thompson[4], Angela Tringali[4], Emily Angell[4], Reed Bowman[4], Sarah W. Fitzpatrick[1,2,3]

Affiliations:

[1]W.K. Kellogg Biological Station, Michigan State University, Hickory Corners, Michigan 49060
[2]Department of Integrative Biology, Michigan State University, East Lansing, Michigan 48824
[3]Ecology, Evolution, and Behavior Program, Michigan State University, East Lansing, Michigan 48824
[4]Archbold Biological Station, Venus, Florida 33960

Correspondence:

To whom correspondence should be addressed: ALL (allewanski@gmail.com)

S1 Supplementary Background and Methods

Table S1. Information on each translocation event including the donor population name, donor name abbreviation used in the paper, the donor population's state (FL = Florida, GA = Georgia), translocation year and month, the number of males and females, and the total number of translocated birds.

Donor population	Donor abbr.	State	Translocation year	Translocation month	Male	Female	Total count
Apalachicola National Forest	ANF	FL	1998	Dec	0	1	1
		FL	2001	Dec	2	2	4
		FL	2003	Nov	3	1	4
		FL	2005	Nov	3	3	6
		FL	2007	Nov	3	3	6
Camp Blanding Joint Training Center	CBJTC	FL	2008	Nov	1	0	1
Fort Benning	FTB	GA	2002	Oct	3	1	4
Fort Stewart	FTS	GA	2009	Oct	5	5	10
		GA	2014	Nov	3	3	6
Osceola National Forest	ONF	FL	2015	Oct	3	3	6
Withlacoochee State Forest—Citrus	WSF-CITRUS	FL	2016	Oct	3	3	6

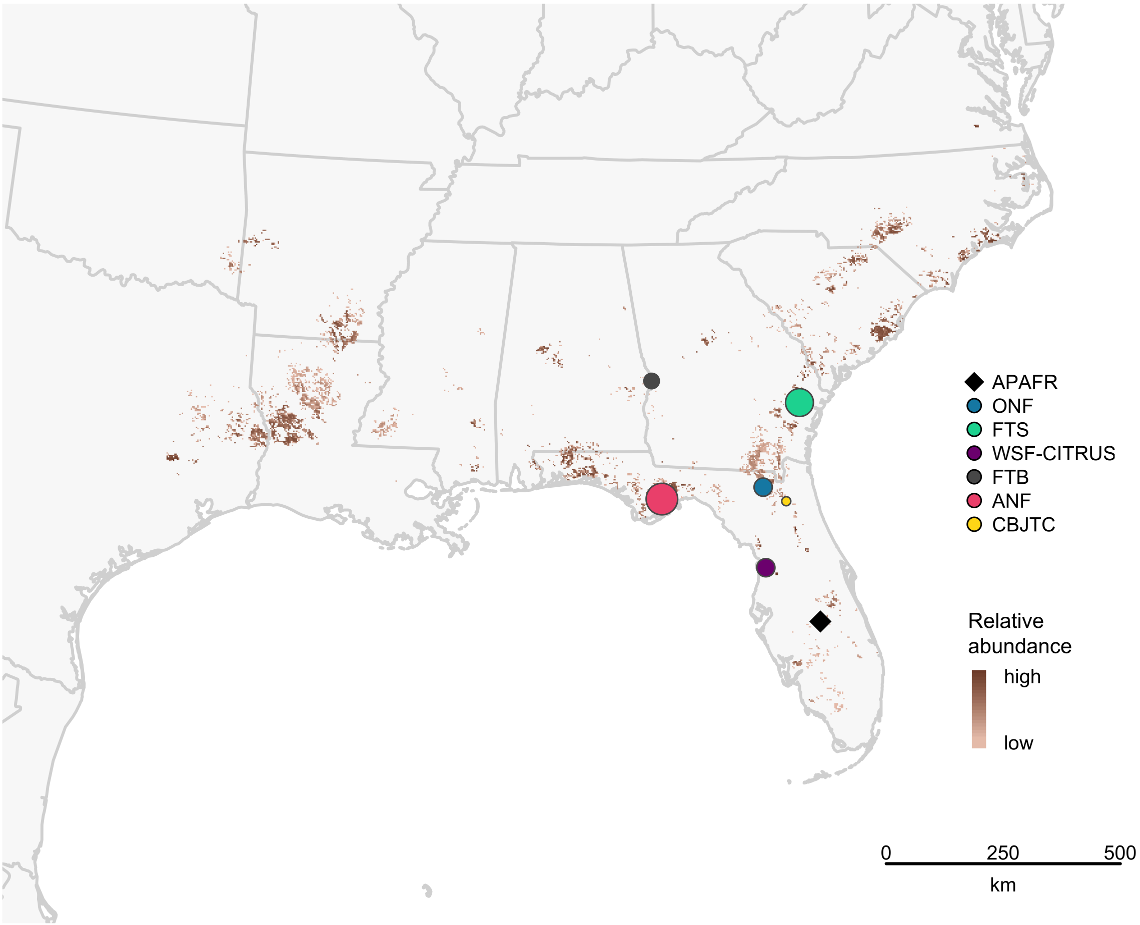

Figure S1. Map of the range-wide distribution of Red-cockaded Woodpeckers and the populations relevant to this project. The distribution of Red-cockaded Woodpecker is represented as eBird's estimate of the species' expected mean relative abundance in 2022 at a 3 km resolution. Darker shades indicate higher relative abundances. The focal population in this study is located at Avon Park Air Force Range (APAFR) in central Florida (visualized as a black diamond). Individuals from six populations distributed across Florida (n = 4) and Georgia (n = 2) have been translocated into the Avon Park population. The size of each population's point approximately scales with number of individuals sourced from the population. The map uses a Lambert Azimuthal Equal Area projection.

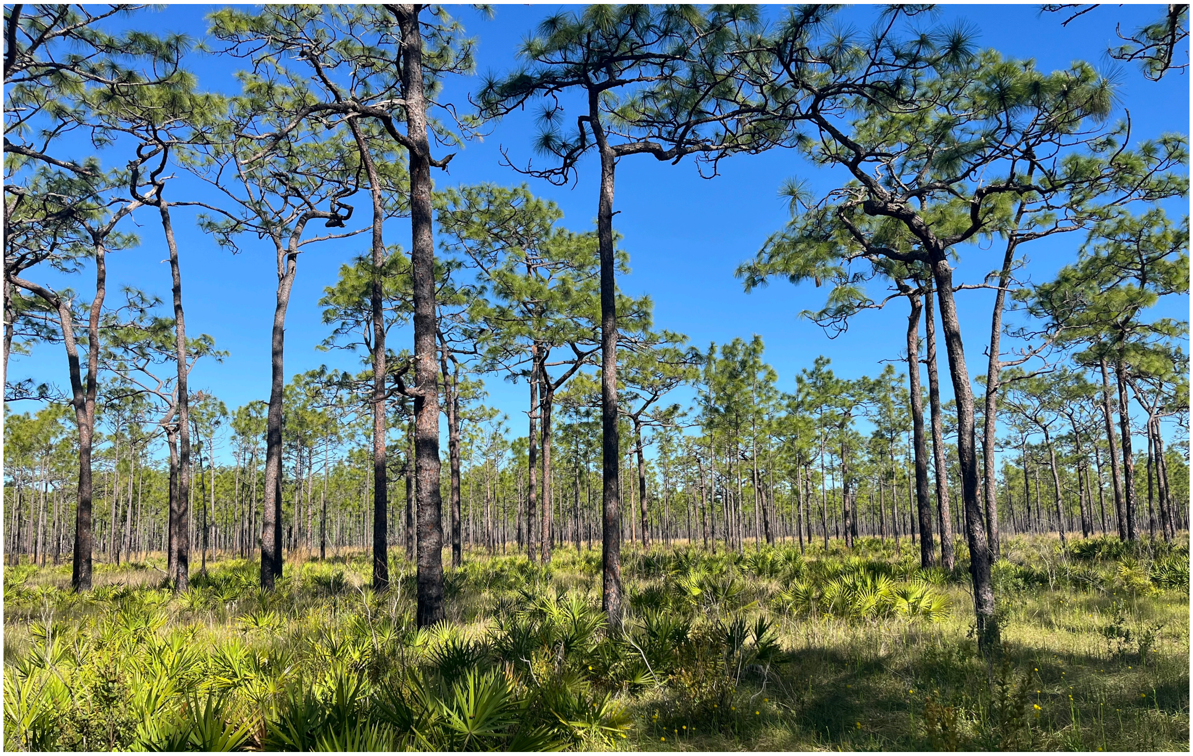

Figure S2. An example of the pine woodland habitat occupied by Red-cockaded Woodpeckers at Avon Park Air Force Range. The primary tree species in these woodlands are longleaf pine (*Pinus palustris*) and South Florida slash pine (*Pinus elliotti var. densa*). The woodlands contain minimal midstories, and their understories are dominated by saw palmetto (*Serenoa repens*) and native grasses such as cutthroat grass (*Panicum abcissum*). The open character of this habitat has been maintained by regular fire from lightning and prescribed burns. Photo taken by AL on 4/5/2024.

Processing and evaluation of census data

Many of the objectives and analyses of this project rely heavily on information regarding each year's population composition, and thus we explored several data processing alternatives and performed multiple data quality evaluations to explore how complete the census data likely were and how data processing choices would influence the dataset used in analyses. As an initial step, we used a capture–mark–recapture modeling framework to estimate the detection probability of birds based on the unprocessed population census data (see S1's *Modeling variation in survival* for further details). We found high estimated probabilities of detection (>0.96; Tables S11 and S13). We thus concluded that nearly all birds in the population were detected in each year's census and that the census data represented an accurate and nearly complete record of population size and composition over the monitoring period.

To further evaluate the census data, we examined the extent of cases where individuals were missing from the census but were detected in preceding and subsequent years. Given the isolated nature of the Avon Park population, these gaps in observations likely represented cases where individuals were missed. However, if individuals were missing for multiple years, this could suggest that birds were leaving the population and then returning. Additionally, the existence of many gaps would suggest that birds were often being missed. We found a total of 38 observation gaps, and all but four of these gaps involved a single year (the remainder all involved two years). Each individual was missed at most once between their first and last census detections. Together, these observations suggest that few birds were missed in the censuses (supporting the survival models) and that birds were not leaving the population for extended durations.

We next processed the census data using four slightly different approaches that varied in the handling of adult and juvenile birds and census gaps:

1. The census data with no further processing.

2. Including both juveniles (birds that were hatched the same year as the census) and adults in the population and filling in any census gaps. The filling of census gaps included adding a record for years where individuals were missed but were detected in both previous and subsequent censuses.

3. Identical to dataset 2 except for cases where the hatch year of a bird was known but it was first detected in a future year's census, we added records for all years starting from and including the hatch year through the first census.

4. Including only adults. Dataset 4 was identical to dataset 3, except that it was limited to adult birds (i.e., individuals were only included in years after their hatch year).

The different processing approaches yielded datasets with similar temporal dynamics in size. The biggest discrepancy stemmed from whether or not the dataset was limited to adults (datasets 1–3 versus dataset 4) with the adult-only dataset (dataset 4) unsurprisingly containing less individuals per year than the other datasets. The numbers of individuals recorded each year were highly correlated across all datasets with all Spearman's ρ values exceeding 0.98 (Fig. S3). We therefore concluded that dataset processing decisions would likely not substantially alter the paper's findings. For all analyses, we used the option 3 dataset (containing both adults and juveniles and filling in likely missing observations starting at hatch year) because this represented the most comprehensive reflection of all individuals existing in the population each year. In the option 3 dataset, across all years, only 1.38% of records were the result of filling in putative missed observations. Per year,

the percentages of records that were filled in ranged from 0% to 4.5% (Fig. S4). A small caveat for the processed dataset is that putative missing observations were unable to be detected in the first (1994) and final (2022) monitoring years, and thus the sizes of the populations in those years may be slightly underestimated.

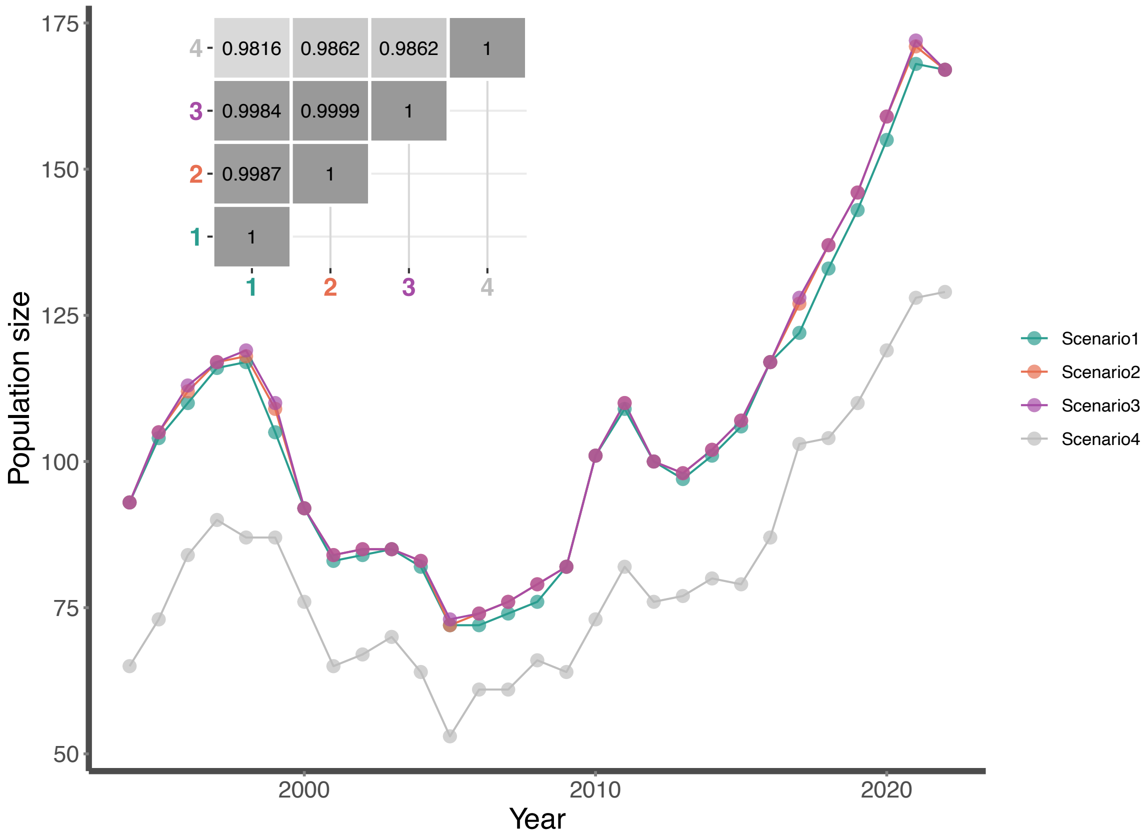

Figure S3. Estimated population sizes in the Red-cockaded Woodpecker population based four different approaches of processing the post-breeding census data. See S1's *Processing and evaluation of census data* section for details on each processing approach. The plot shows the population size for each monitoring year based on each processing approach. The grid in the plot's upper left shows the correlations in population size between each of processing approaches.

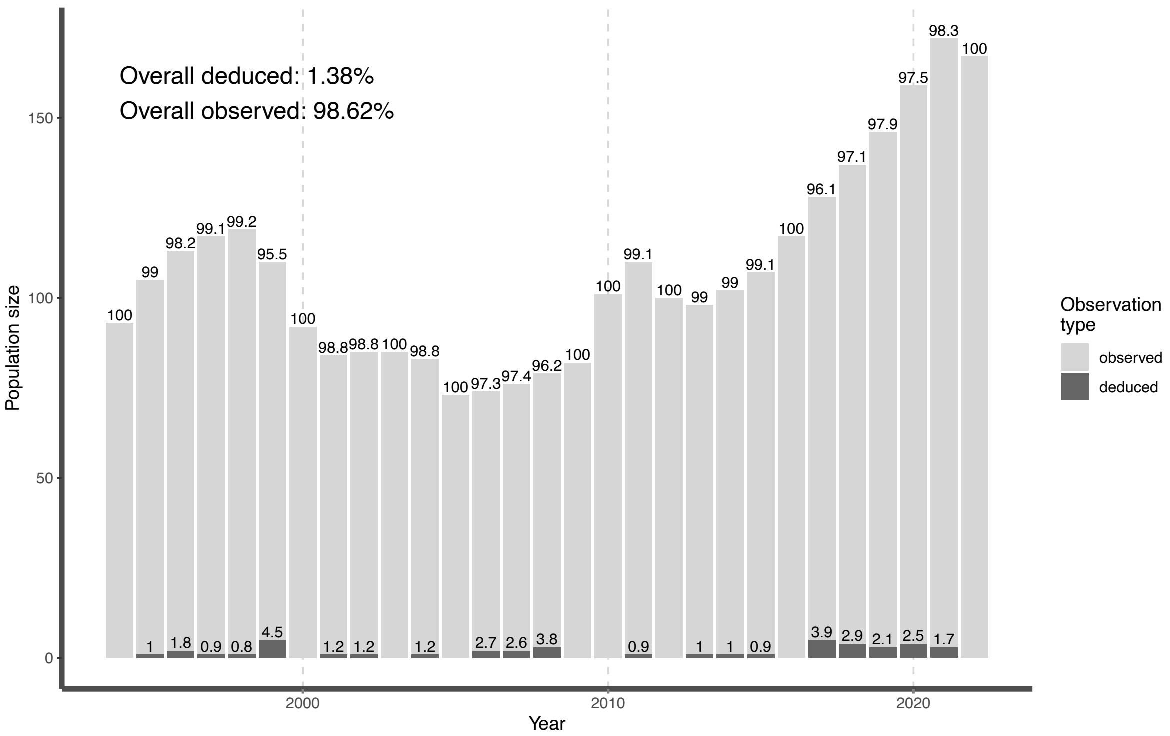

Figure S4. The population composition dataset used in the paper (the option 3 dataset) includes observations that either represent true observations of birds (*observed*) or observations that are deduced based on true observations from nearby years. The plot shows the proportions of observations that are observed versus deduced in the processed population composition dataset.

Modeling variation in survival

We generated capture histories for all uniquely banded individuals in the population from 1994–2022 based on whether they were observed in the annual post-breeding census. We removed 23 individuals for which sex was unknown. We had two main questions about how survival varies in the population and subset the capture histories accordingly. First, we were interested in whether survival varied based on an individual's degree of translocation ancestry. We limited this analysis to locally hatched birds to focus on the effects of translocations beyond the direct performance of the translocated birds. Second, we were interested in survival differences between directly translocated individuals that established in the population (n = 38) compared to resident birds with 0% translocation ancestry (n = 401).

For each dataset, we fit a set of models that varied in their assumptions of apparent survival (φ) and recapture probability (p) based on Cormack–Jolly–Seber (CJS) method (1–3) in `Program MARK` using the `RMark` interface in `R` version 4.3.0 (4, 5). We fit similar sets of models for both datasets that included all combinations of time, sex, and a translocation variable, including two- and three-way interactions for survival and time-dependent and constant models for recapture probabilities. The difference between the model sets was that in the first analysis, the translocation variable representing an individual's degree of translocation ancestry (ranging between 0–1), while in the second analysis, the translocation variable was categorical and indicated whether or not a bird was translocated. We used a maximum likelihood approach to fit the models and compared among them using Akaike's Information Criterion adjusted for sample size (AICc) and AICc weights (6). For both analyses we obtained maximum likelihood estimates of parameters from the best-supported model. We tested for overdispersion of the datasets using the Fletcher c-hat estimator and found that there was little (c-hat = 1; (7)). Detection probability was high and did not vary by time ($p \geq 0.97$), allowing precise estimation of our main parameter of interest, annual survival.

Models of reproductive success

We had two primary objectives for our analyses of short-term fitness. First, we evaluated whether the translocated breeders showed differences in lifetime reproductive success compared to non-translocated breeders in the Avon Park population (hereafter *translocation analyses*). Second, beyond the translocated birds, we evaluated whether genetic material inherited from translocated birds was associated with differences in several reproductive success measures, including lifetime reproductive success, total number of nesting years, and mean annual reproductive success (hereafter *translocation ancestry analyses*). Together, these two sets of analyses helped establish whether the translocations were associated with reproductive improvements in the population's breeders. We estimated all models in a Bayesian framework with NUTS Hamiltonian Monte Carlo using `stan` (8) via the `R` package `brms` (9). For all models, we used four chains of 10,000 iterations each and a thinning rate of 1. We used diffuse priors for the parameters of the models. For the parameters associated with the fixed effects predictors, we used normal distributions with mean = 0 and sd = 5. For all other parameters, we used `stan` defaults. Below, we provide further details on our modeling approach and the evaluation and visualization of modeling results.

Translocation analyses

For the translocation models, we limited our analyses to translocated breeders and non-translocated breeders hatched during the monitoring period that had no expected ancestry from translocated birds. Excluding non-translocated descendants of translocated birds helped isolate any potential differences in reproductive success between translocated birds and Avon Park breeders that were unaffected by the translocations. Although the translocated birds did not hatch at Avon Park, their lifetime reproductive success values were still directly comparable with the locally hatched birds because they were translocated in the fall following their hatching. Thus, none of their lifetime reproductive success could have accrued before their time at Avon Park.

We carried out the translocation analyses in two stages. First, we fit a series of models involving different combinations of the focal variable [whether or not the bird was translocated (*translocation status*)] and a set of potentially relevant covariates. Once we identified the top performing predictors, we used the model with these predictors to examine the effects of translocation status. If translocation status was not in the set of best predictors, we examined the effects of translocation status in the top performing model that included this variable. Beyond translocation status, we considered three candidate predictors: first breeding year, mean group size, and sex. First breeding year represents the calendar year that an individual first breeds and could relate to lifetime reproductive success if reproductive success varied through time. Mean group size was calculated as the mean size of the breeding groups to which an individual belonged during all of its nesting events. This variable was included because a well-established relationship exists between reproductive success of a breeding group and its size. Sex indicates whether an individual is male or female. Sex may be important because male and female Red-cockaded Woodpeckers can display different reproductive strategies, which could translate to differences in reproductive success. For example, early in life, males regularly (but not exclusively) remain at their natal cluster as helpers while females generally disperse away from their natal cluster in search of breeding vacancies. Helpers may wait longer than those that disperse to become breeders but the dispersing birds often suffer higher mortality rates (10). For modeling, we shifted first breeding year so that the earliest year was recorded as zero, and for sex, we used male as the reference level.

We created models with all combinations and subsets of the candidate predictors. Models were

fit with the candidate predictors as fixed effects (all as main effects) and a random effect term based on the genetic additive relationship matrix calculated from the population's pedigree to account for variation in relatedness across individuals. Given that lifetime reproductive success was a count variable, we used a Poisson distribution with log link for all models. Another consideration for these models is that some of the breeders were still alive at the end of the monitoring and thus their lifetime reproductive success was potentially not fully realized. To address this, we treated the living individuals as right-censored observations in the model. The model syntax took the following form:

```
lifetime repro. success | cens(censored) ~ predictors + (1|gr(id,cov=A)).
```

In this line of code, `lifetime repro. success` is the predictor, `cens(censored)` is the term used to censor the observations involving living individuals, `predictors` represents the set of one or more fixed effects, and the `(1|gr(id,cov=A))` is the term used to input the additive genetic relationship matrix of the individuals as a random effect.

We ranked the models using expected log pointwise predictive density (ELPD) that we estimated with leave-one-out cross-validation, which we calculated with the `R` package `loo (11, 12)`. Higher ELPD values are associated with better performing models. The results of this model comparison are shown in Table S7. The top performing model (highest ELPD value) was the model that contained all predictors: translocation status, mean group size, first breeding year, and sex (Table S2).

Translocation ancestry analyses

For the translocation ancestry models, we limited our analyses to breeders that were hatched at Avon Park during the monitoring period, and thus all translocated birds (and other pedigree founders) were excluded from these analyses. By only considering locally hatched birds, we aimed to examine the extended demographic impacts of translocations beyond the direct effects of the translocations.

For the translocation ancestry analyses, we considered three reproductive success measures: mean annual reproductive success, total nesting years, and lifetime reproductive success. Examining these variables together may help reveal whether translocation ancestry (and the other predictors) influenced lifetime reproductive success and how exactly these relationships emerged. For example, it is possible that the relationship between a predictor and lifetime reproductive success emerged because the predictor had an effect on the total number of years an individual was reproductively active, the average success that an individual had each year it nested, or both. For these models, we considered six candidate predictors: translocation ancestry, ancestry count, first breeding year, mean group size, sex, and F_p. The first two predictor variables, translocation ancestry and ancestry count, represented the variables describing translocation-related attributes of individuals. Translocation ancestry is the expected proportion of an individual's ancestry that originates from a translocation donor population. Ancestry count was the number of pedigree founder groups (i.e., the six donor populations and the non-translocated pedigree founders) from which an individual is expected to inherit genetic material, and represents a simple quantification of admixture. The latter four variables represent other covariates that could plausibly have an impact on reproductive success. These predictors are identical to those included in the translocation with the addition of F_P because we were interesting in examining whether inbreeding was associated

with any reproductive consequences and because it has an established connection to reproductive success.

We used the following workflow for the lifetime reproductive success and total nesting years models. First, we fit a series of models with all combinations of predictors (only considering main effects) using the following general syntax:

```
repro. success measure | cens(censored) ~ predictors + (1|gr(id,cov=A)).
```

We evaluated model performance using ELPD and identified all predictors in the set of models possessing ELPD values within 4 of the top model (an ELPD difference of 4 is a rule of thumb for identifying models of comparable performance (13)). We then used the model with all predictors included in the competitive models to interpret the effects of each predictor. We note that for each reproductive measure's model set, the model containing all predictors found across competitive models had an ELPD difference of less than 4 compared to the top model. For both the lifetime reproductive success and total nesting years models, we used a Poisson distribution with log link, we right-censored individuals that were still alive in the final monitoring year, and we included the genetic additive relationship matrix as a random effect.

Mean annual reproductive success is a continuous variable that cannot be negative but can include zero. Thus, we used a hurdle model with a hurdle_gamma distribution. The model contained two components. The hurdle component models whether or not birds have a mean fledgling value greater than zero. The second component models the effects of the predictors for values of the response that are greater than zero. We used the general `brms` syntax to fit the mean annual reproductive success models:

```
mean annual reproductive success | cens(censored) ~ predictors + (1|gr(id,cov=A))

hurdle ~ predictors + (1|gr(id,cov=A)).
```

We analyzed the predictors of mean annual reproductive success in two stages. First, we fit a series of models with all combinations of predictors in the non-zero component of the model and limited the hurdle component to only include an intercept. Similar to the workflow for the other reproductive measures, we identified the predictors found across the competitive models (ELPD difference of less than 4 relative to the top model). We then fit an additional set of models to examine the predictors of the hurdle component. For this second model set, we included the previously identified predictors of the non-zero component but varied the predictors of the hurdle component. To limit model complexity we only included one predictor at a time for the hurdle component. We then compared the performance of this second model set using ELPD. For all mean annual reproductive success models, we included the genetic additive relationship matrix as a random effect in both components. We right-censored the observations associated with living individuals in the second component. However, `brms` does not currently support censoring for the hurdle component, and thus the living individuals were not censored.

Model visualization

To visualize the models, we used the `add_epred_draws` function from the `tidy_bayes` (14) package (re_formula argument set to NA) to draw values for the expectation of the posterior predictive distribution. When visualizing the estimated relationship of a continuous variable (i.e., translocation ancestry in the translocation ancestry models), we drew 500 expected values based on a

new dataset with 100 values equally spaced between the lowest and highest values in the observed dataset. For the new dataset, we set the non-focal, continuous predictors in the model to their median value in the observed dataset, and we set sex to female. We calculated the 95% credible interval for the relationship by identifying the 2.5% and 97.5% percentiles at each of the 100 values. When visualizing the estimated relationship of a binary variable (i.e., translocation status in the translocation models), we drew 500 expected values based on a new dataset that included both levels of the variable, the non-focal continuous variables set to their median value in the observed dataset, and sex set to female.

Model evaluation

We took several steps to evaluate the models after they were fit. First, we checked model convergence with $\hat{R}$ values. Across all models, $\hat{R}$ values were near one and all were $\leq$1.003, providing evidence that the models converged. Additionally, the bulk and tail effective sample sizes for all parameters suggested that sufficient sampling took place to reliably estimate the parameters. To evaluate model fit, we performed posterior predictive checks for the final models that were used for interpreting the effects of the predictors (Figs. S5 and S6) by comparing the distribution of the observed data to the distribution of values drawn from the posterior predictive distribution. We performed the checks based on 1,000 draws from the posterior distributions with the `pp_check` function from the `brms` package.

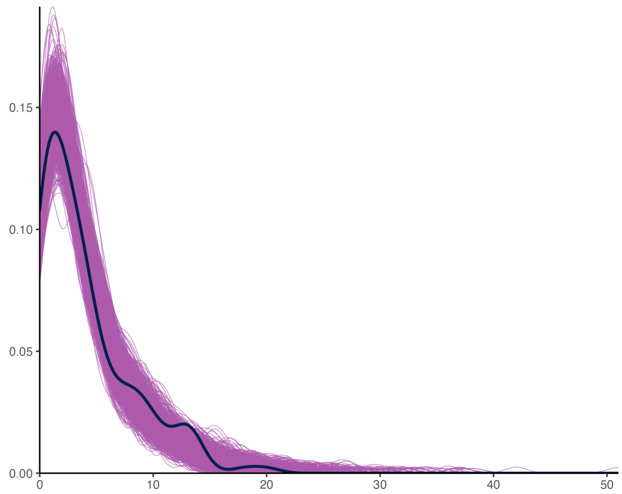

Figure S5. Posterior predictive check for the top fitting translocation model that was examining differences in lifetime reproductive success between locally hatched breeders with no translocation ancestry and translocated birds.

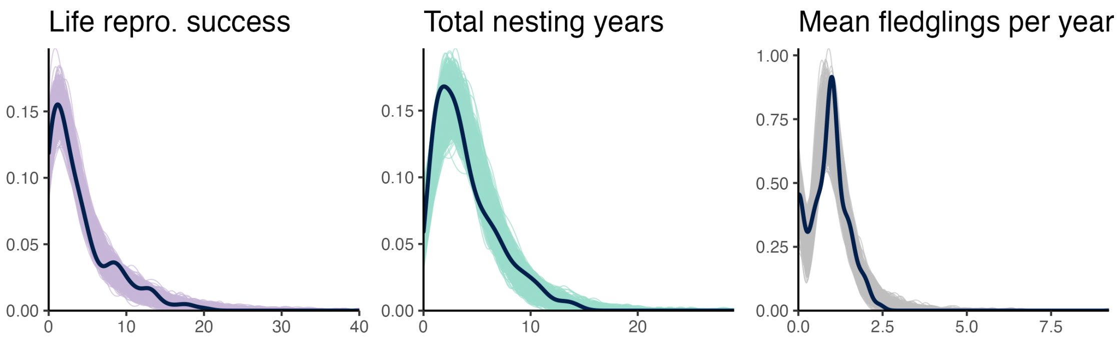

Figure S6. Posterior predictive checks for the top fitting translocation ancestry model examining the predictors of lifetime reproductive success and auxiliary models examining the predictors of total nesting years and mean fledglings per year based on a dataset that was limited to breeders that were hatched at Avon Park.

S2 Supplementary Results

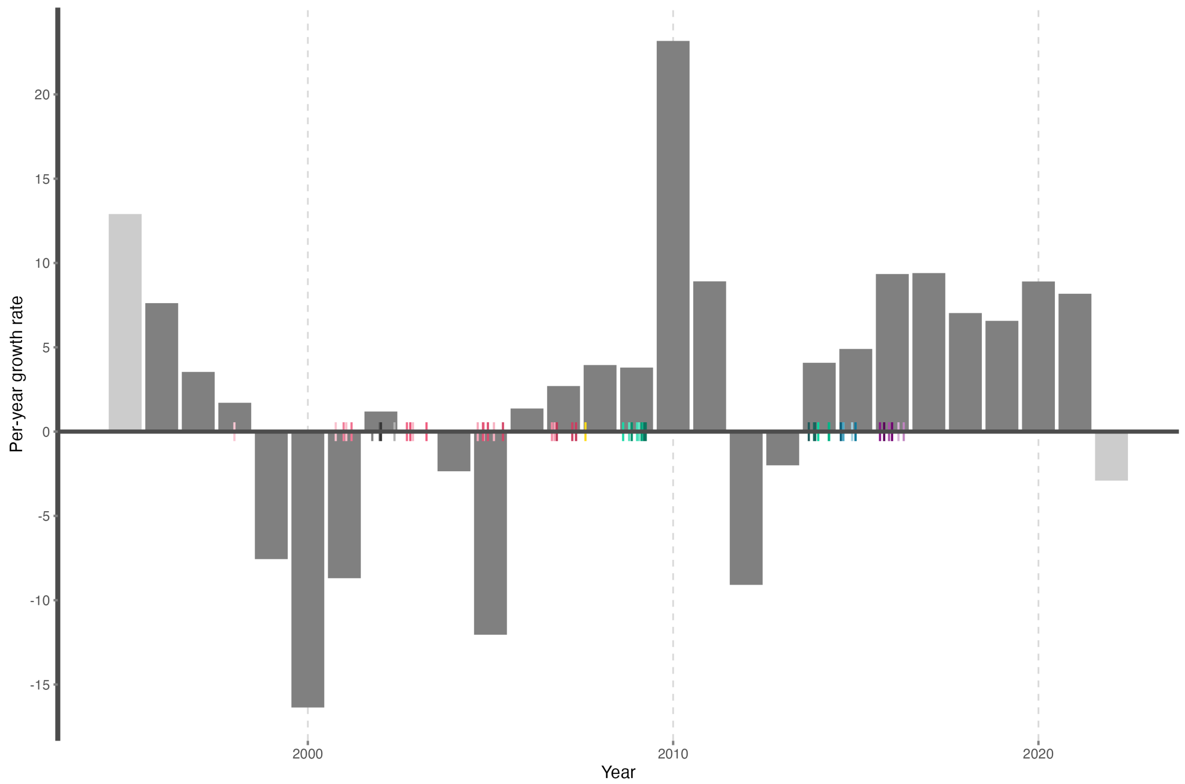

Figure S7. Inter-year percentage changes in population size over the monitoring period. There is likely a slight inflation and deflation of values associated with the last and final years, respectively. These distortions arose because there are likely a handful of individuals that are alive in the population in the first and last monitoring years that were missed in the censuses. Our census processing approach attempted to deduce these missing observations by identifying years where individuals were missed but were observed in earlier and later years. However, this approach is not possible for the first and last monitoring years and so these population sizes are likely more underestimated than the other years. Consequently, comparing the second year population size to the first will artificially increase the percentage change compared to a situation where the first year's population was not underestimated. Conversely, comparing the last year to the penultimate year will artificially decrease the percentage change compared to a situation where the last year's population was not underestimated. The timing of translocations are shown along the horizontal axis.

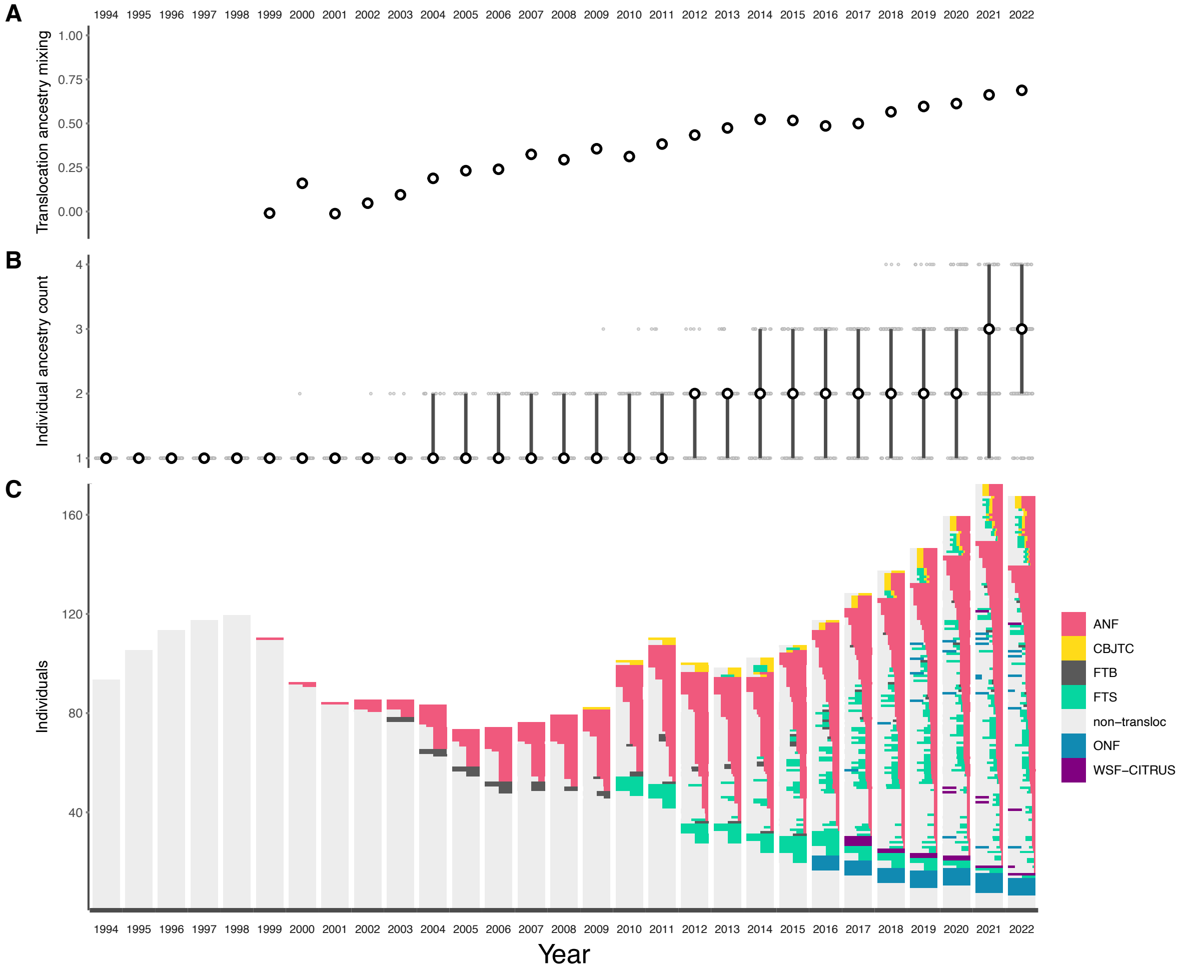

Figure S8. Individual-level ancestry composition of the APAFR population. (A) Quantification of the degree of individual-level mixing of each pedigree founder group's ancestry using the m_d metric. When $m_d = 0$, there is no mixing of ancestry (all individuals have ancestry from one source) and a value of one indicates that there is equal mixing of ancestry across all individuals. When only the non-translocation ancestry exists in the population, the mixing parameter is undefined, which is why no value exists before 1999 when no translocation ancestry existed in the population (B) A summary of how many sources of ancestry that individuals possessed based on each translocation donor population and non-translocation ancestry. For example, if an individual had ancestry from non-translocated ancestors and also ancestors from two different translocation donor populations, it would have an ancestry count of three. The large, black points represent the median count value across individuals while the vertical bars show the 10th and 90th percentiles of count values. The raw count values for individuals are shown as small, gray points. (C) Visual illustration of the expected ancestry of each individual from non-translocated ancestors and ancestors from each translocation donor population. These proportions were calculated as the frequency of alleles that each individual inherits from each pedigree founder group based on the gene drop simulations

Table S2. Overview of model fit for the model set examining the relationship between whether or not a bird was translocated and lifetime reproductive success (as well as other predictors). For each model, the table includes its rank based on expected log pointwise predictive density (ELPD), its fixed effects, the difference in ELPD compared to the top model (*ELPD diff*), and the standard error of the ELPD difference (*SE ELPD diff*).

Rank	Fixed effects	ELPD diff	SE ELPD diff
1	Mean group size + First breeding year + Sex + transloc	0.000	0.000
2	Mean group size + First breeding year + transloc	-1.294	2.045
3	First breeding year + transloc	-3.770	6.758
4	First breeding year + Sex + transloc	-3.795	6.435
5	Mean group size + First breeding year + Sex	-5.803	2.942
6	Mean group size + First breeding year	-7.854	3.174
7	First breeding year + Sex	-9.403	7.065
8	Mean group size + transloc	-9.434	3.588
9	Mean group size + Sex + transloc	-9.598	3.151
10	Mean group size	-10.140	3.705
11	Mean group size + Sex	-10.176	3.448
12	First breeding year	-12.097	7.101
13	Sex	-15.807	8.459
14	Sex + transloc	-15.930	8.206
15	transloc	-17.535	8.660

Table S3. Parameter estimates for the model built to examine the relationship between whether or not a bird was translocated and lifetime reproductive success. The table includes information on the parameters included in the model, and the following information for each parameter: estimate, lower and upper 95% credible intervals, $\hat{R}$ (Rhat), bulk effective sample size (ESS), and tail ESS. If not indicated with *[rand. effect]*, the parameter represents a fixed effect in the model.

Response	Parameter	Estimate	lower 95% CI	upper 95% CI	Rhat	Bulk ESS	Tail ESS
lifetime repro. success	Intercept	-0.745	-1.596	0.099	1	16598.558	15555.24
	Mean group size	0.812	0.515	1.113	1	17357.328	15721.28
	First breeding year	-0.056	-0.092	-0.021	1	14361.450	14258.62
	Sex (female)	-0.253	-0.589	0.070	1	16037.239	15141.45
	Translocated	0.984	0.407	1.586	1	11130.638	12950.60
	sd(Intercept) [rand. effect]	0.966	0.785	1.175	1	7749.323	12581.68

Table S4. Overview of model fit for the model set examining the predictors of total nesting years. For each model, the table includes its rank based on expected log pointwise predictive density (ELPD), its fixed effects, the difference in ELPD compared to the top model (*ELPD diff*), and the standard error of the ELPD difference (*SE ELPD diff*).

Rank	Fixed effects	ELPD diff	SE ELPD diff
1	Mean group size + First breeding year + Sex + Transloc. ancestry + Ancestry count	0.000	0.000
2	Mean group size + First breeding year + Sex + Transloc. ancestry + Fped	-0.462	1.420
3	Mean group size + First breeding year + Sex + Transloc. ancestry + Ancestry count + Fped	-0.702	1.174
4	Mean group size + First breeding year + Transloc. ancestry + Ancestry count + Fped	-0.885	2.102
5	Mean group size + First breeding year + Sex + Transloc. ancestry	-1.158	1.197
6	Mean group size + First breeding year + Transloc. ancestry + Ancestry count	-1.336	1.846
7	Mean group size + First breeding year + Transloc. ancestry	-1.829	1.920
8	Mean group size + First breeding year + Transloc. ancestry + Fped	-2.065	2.175
9	Mean group size + First breeding year + Sex + Ancestry count + Fped	-6.424	3.103
10	Mean group size + First breeding year + Sex + Ancestry count	-7.438	2.899
11	Mean group size + First breeding year + Ancestry count	-7.729	3.733
12	Mean group size + First breeding year + Sex	-8.393	3.712
13	Mean group size + First breeding year + Ancestry count + Fped	-9.240	3.923
14	Mean group size + First breeding year + Sex + Fped	-9.361	3.688
15	Mean group size + First breeding year	-10.948	4.466
16	Mean group size + First breeding year + Fped	-11.024	4.551
17	First breeding year + Sex + Transloc. ancestry + Ancestry count + Fped	-12.883	5.643
18	First breeding year + Sex + Transloc. ancestry	-13.061	5.934
19	First breeding year + Sex + Transloc. ancestry + Ancestry count	-13.660	5.713
20	First breeding year + Transloc. ancestry + Ancestry count	-13.663	5.959
21	First breeding year + Sex + Transloc. ancestry + Fped	-13.688	5.855
22	First breeding year + Transloc. ancestry + Ancestry count + Fped	-13.825	6.047
23	Mean group size + Sex + Ancestry count	-14.338	4.523
24	Mean group size + Sex + Transloc. ancestry + Ancestry count + Fped	-14.534	4.431
25	Mean group size + Sex + Ancestry count + Fped	-14.537	4.560
26	First breeding year + Transloc. ancestry	-14.539	6.175
27	Mean group size + Sex + Transloc. ancestry + Ancestry count	-14.549	4.288
28	Mean group size + Transloc. ancestry + Ancestry count	-15.306	4.549
29	First breeding year + Transloc. ancestry + Fped	-15.529	6.279
30	Mean group size + Transloc. ancestry + Ancestry count + Fped	-15.572	4.723
31	Mean group size + Ancestry count	-15.891	4.895
32	Mean group size + Ancestry count + Fped	-15.918	5.033
33	First breeding year + Sex + Ancestry count	-21.505	6.856
34	First breeding year + Sex + Ancestry count + Fped	-21.584	6.623
35	First breeding year + Ancestry count	-22.302	7.105
36	First breeding year + Sex	-23.225	7.138
37	First breeding year + Ancestry count + Fped	-23.377	6.902
38	First breeding year + Sex + Fped	-24.094	7.157
39	First breeding year + Fped	-25.666	7.505
40	First breeding year	-26.370	7.853
41	Mean group size + Sex + Fped	-29.400	6.894
42	Mean group size	-29.824	7.199
43	Mean group size + Sex	-30.048	7.035
44	Sex + Transloc. ancestry + Ancestry count + Fped	-30.401	7.763
45	Mean group size + Sex + Transloc. ancestry + Fped	-30.607	6.858
46	Mean group size + Transloc. ancestry + Fped	-30.634	7.039
47	Mean group size + Transloc. ancestry	-30.669	7.324
48	Sex + Transloc. ancestry + Ancestry count	-30.710	7.812
49	Transloc. ancestry + Ancestry count	-30.712	7.955
50	Sex + Ancestry count	-30.723	7.939
51	Mean group size + Sex + Transloc. ancestry	-31.023	7.151
52	Mean group size + Fped	-31.204	7.274
53	Ancestry count	-31.274	8.215
54	Ancestry count + Fped	-31.359	8.030
55	Transloc. ancestry + Ancestry count + Fped	-31.441	7.945
56	Sex + Ancestry count + Fped	-32.247	7.901
57	Fped	-48.855	10.707
58	Sex	-48.860	10.637
59	Transloc. ancestry	-49.810	10.836
60	Sex + Transloc. ancestry	-50.174	10.787
61	Transloc. ancestry + Fped	-50.505	10.572
62	Sex + Transloc. ancestry + Fped	-50.570	10.446
63	Sex + Fped	-50.733	10.949

Table S5. Overview of model fit for the model set examining the predictors of mean annual reproductive success. For each model, the table includes its rank based on expected log pointwise predictive density (ELPD), its fixed effects, the difference in ELPD compared to the top model (*ELPD diff*), and the standard error of the ELPD difference (*SE ELPD diff*).

Rank	Fixed effects	ELPD diff	SE ELPD diff
1	Mean group size + First breeding year	0.000	0.000
2	Mean group size + First breeding year + Ancestry count	-0.515	0.215
3	Mean group size + First breeding year + Transloc. ancestry	-0.528	0.398
4	Mean group size + First breeding year + Fped	-0.545	1.086
5	Mean group size + First breeding year + Sex	-0.938	0.208
6	Mean group size + Transloc. ancestry	-0.971	1.640
7	Mean group size + First breeding year + Sex + Transloc. ancestry	-1.300	0.405
8	Mean group size + First breeding year + Transloc. ancestry + Fped	-1.389	1.089
9	Mean group size + First breeding year + Ancestry count + Fped	-1.509	1.115
10	Mean group size + First breeding year + Transloc. ancestry + Ancestry count	-1.546	0.439
11	Mean group size + First breeding year + Sex + Fped	-1.559	1.099
12	Mean group size + First breeding year + Sex + Ancestry count	-1.768	0.240
13	Mean group size	-1.783	2.128
14	Mean group size + Transloc. ancestry + Ancestry count	-1.810	1.373
15	Mean group size + Ancestry count	-1.928	1.613
16	Mean group size + Sex + Transloc. ancestry	-2.076	1.649
17	Mean group size + Sex	-2.077	2.120
18	Mean group size + First breeding year + Sex + Transloc. ancestry + Fped	-2.186	1.119
19	Mean group size + First breeding year + Sex + Transloc. ancestry + Ancestry count	-2.274	0.466
20	Mean group size + First breeding year + Sex + Ancestry count + Fped	-2.350	1.119
21	Mean group size + Transloc. ancestry + Fped	-2.403	1.831
22	Mean group size + First breeding year + Transloc. ancestry + Ancestry count + Fped	-2.439	1.124
23	Mean group size + Ancestry count + Fped	-2.474	1.854
24	Mean group size + Fped	-2.582	2.331
25	Mean group size + Sex + Ancestry count	-2.635	1.598
26	Mean group size + Sex + Transloc. ancestry + Ancestry count	-2.814	1.379
27	Mean group size + Sex + Ancestry count + Fped	-3.117	1.868
28	Mean group size + Transloc. ancestry + Ancestry count + Fped	-3.144	1.606
29	Mean group size + First breeding year + Sex + Transloc. ancestry + Ancestry count + Fped	-3.245	1.129
30	Mean group size + Sex + Transloc. ancestry + Ancestry count + Fped	-3.629	1.631
31	Mean group size + Sex + Fped	-3.744	2.336
32	First breeding year	-16.313	5.684
33	First breeding year + Sex	-17.134	5.691
34	First breeding year + Transloc. ancestry	-17.161	5.745
35	First breeding year + Fped	-17.244	5.823
36	First breeding year + Ancestry count	-17.424	5.691
37	Ancestry count	-17.539	5.942
38	Transloc. ancestry	-17.819	6.252
39	First breeding year + Transloc. ancestry + Fped	-17.962	5.864
40	First breeding year + Transloc. ancestry + Ancestry count	-18.057	5.752
41	First breeding year + Sex + Ancestry count	-18.091	5.691
42	First breeding year + Sex + Transloc. ancestry	-18.091	5.750
43	First breeding year + Ancestry count + Fped	-18.176	5.823
44	Transloc. ancestry + Ancestry count	-18.392	5.977
45	First breeding year + Sex + Fped	-18.470	5.831
46	Sex + Transloc. ancestry	-18.639	6.267
47	First breeding year + Sex + Ancestry count + Fped	-18.744	5.826
48	Ancestry count + Fped	-18.778	6.031
49	First breeding year + Sex + Transloc. ancestry + Fped	-18.899	5.873
50	First breeding year + Sex + Transloc. ancestry + Ancestry count	-18.926	5.761
51	Sex	-18.950	6.374
52	Transloc. ancestry + Ancestry count + Fped	-18.956	6.048
53	Transloc. ancestry + Fped	-19.029	6.352
54	First breeding year + Transloc. ancestry + Ancestry count + Fped	-19.070	5.866
55	Sex + Transloc. ancestry + Ancestry count	-19.204	5.992
56	First breeding year + Sex + Transloc. ancestry + Ancestry count + Fped	-19.877	5.847
57	Sex + Ancestry count + Fped	-19.996	6.035
58	Sex + Fped	-20.054	6.514
59	Sex + Transloc. ancestry + Fped	-20.287	6.352
60	Sex + Transloc. ancestry + Ancestry count + Fped	-20.367	6.058
61	Mean group size + Sex + Transloc. ancestry + Fped	-103.577	16.665
62	Fped	-184.664	25.514
63	Sex + Ancestry count	-257.537	34.124

Table S6. Overview of model fit for the model set examining the predictors of mean annual reproductive success with differing predictors in the hurdle component. All models were constructed with the identical set of predictors for the non-zero component: Mean group size + First breeding year + Sex + Transloc. ancestry + Ancestry count + Fped. These predictors represented the set of predictors found in competitive models (ELPD diff < 4) involving an intercept-only hurdle term. The models shown here only differ in the term included in the hurdle term. For each model, the table includes its rank based on expected log pointwise predictive density (ELPD), the predictor in the model's hurdle component, its fixed effects, the difference in ELPD compared to the top model (*ELPD diff*), and the standard error of the ELPD difference (*SE ELPD diff*).

Rank	Hurdle term	ELPD diff	SE ELPD diff
1	Mean group size	0.0000000	0.000000
2	Intercept only	-0.4459256	2.590916
3	Sex	-0.5882669	3.090595
4	Fped	-0.6660266	3.253929
5	First breeding year	-1.0553330	2.568861
6	Ancestry count	-1.2207388	2.639992
7	Translocation ancestry	-1.2304886	2.855183

Table S7. Overview of model fit for the model set examining the predictors of lifetime reproductive success. For each model, the table includes its rank based on expected log pointwise predictive density (ELPD), its fixed effects, the difference in ELPD compared to the top model (*ELPD diff*), and the standard error of the ELPD difference (*SE ELPD diff*).

Rank	Fixed effects	ELPD diff	SE ELPD diff
1	Mean group size + First breeding year + Sex + Transloc. ancestry + Ancestry count	0.000	0.000
2	Mean group size + First breeding year + Sex + Transloc. ancestry + Ancestry count + Fped	-0.823	1.341
3	Mean group size + First breeding year + Sex + Transloc. ancestry	-2.308	2.175
4	Mean group size + First breeding year + Sex + Transloc. ancestry + Fped	-2.504	2.517
5	Mean group size + First breeding year + Transloc. ancestry + Ancestry count + Fped	-2.726	2.230
6	Mean group size + First breeding year + Transloc. ancestry + Fped	-3.389	3.026
7	Mean group size + First breeding year + Transloc. ancestry	-3.646	2.864
8	Mean group size + First breeding year + Transloc. ancestry + Ancestry count	-4.050	2.292
9	First breeding year + Sex + Transloc. ancestry + Ancestry count	-4.714	7.623
10	First breeding year + Sex + Transloc. ancestry + Ancestry count + Fped	-5.693	7.729
11	First breeding year + Sex + Transloc. ancestry	-7.333	7.868
12	First breeding year + Transloc. ancestry + Ancestry count	-7.972	7.649
13	First breeding year + Sex + Transloc. ancestry + Fped	-8.012	7.709
14	First breeding year + Transloc. ancestry + Fped	-8.029	7.952
15	First breeding year + Transloc. ancestry + Ancestry count + Fped	-8.169	7.748
16	Mean group size + First breeding year + Sex + Ancestry count	-8.673	3.212
17	First breeding year + Transloc. ancestry	-8.947	8.127
18	Mean group size + First breeding year + Sex + Ancestry count + Fped	-11.268	3.453
19	Mean group size + First breeding year + Ancestry count + Fped	-12.598	3.860
20	Mean group size + First breeding year + Ancestry count	-13.137	3.907
21	Mean group size + First breeding year + Sex	-13.602	4.576
22	First breeding year + Sex + Ancestry count + Fped	-14.144	7.963
23	First breeding year + Sex + Ancestry count	-14.156	7.972
24	First breeding year + Ancestry count	-14.874	8.145
25	Mean group size + First breeding year + Sex + Fped	-14.945	4.845
26	Mean group size + Sex + Transloc. ancestry + Ancestry count + Fped	-15.450	3.992
27	Mean group size + First breeding year	-17.588	5.072
28	First breeding year + Ancestry count + Fped	-17.590	8.139
29	First breeding year + Sex	-17.867	8.232
30	Mean group size + Sex + Transloc. ancestry + Ancestry count	-18.055	4.046
31	First breeding year + Sex + Fped	-18.489	8.525
32	Mean group size + Transloc. ancestry + Ancestry count	-18.580	4.310
33	Mean group size + Sex + Ancestry count + Fped	-19.652	4.327
34	Mean group size + First breeding year + Fped	-19.673	4.760
35	Mean group size + Sex + Ancestry count	-20.146	4.409
36	First breeding year	-20.166	8.946
37	Mean group size + Transloc. ancestry + Ancestry count + Fped	-20.778	4.618
38	Mean group size + Ancestry count + Fped	-21.261	4.566
39	First breeding year + Fped	-21.549	8.520
40	Mean group size + Ancestry count	-22.363	4.817
41	Sex + Ancestry count + Fped	-24.019	9.195
42	Sex + Ancestry count	-24.946	9.194
43	Transloc. ancestry + Ancestry count	-26.747	9.587
44	Sex + Transloc. ancestry + Ancestry count	-26.917	9.443
45	Ancestry count	-26.930	9.547
46	Sex + Transloc. ancestry + Ancestry count + Fped	-27.156	9.567
47	Ancestry count + Fped	-27.764	9.380
48	Transloc. ancestry + Ancestry count + Fped	-29.910	9.713
49	Mean group size + Sex + Transloc. ancestry	-38.020	7.537
50	Mean group size + Sex + Fped	-38.064	7.216
51	Mean group size + Transloc. ancestry	-38.920	7.518
52	Mean group size + Transloc. ancestry + Fped	-39.097	7.198
53	Mean group size + Sex + Transloc. ancestry + Fped	-39.206	7.240
54	Mean group size + Sex	-39.292	7.343
55	Mean group size + Fped	-40.492	7.419
56	Mean group size	-41.344	7.733
57	Sex + Transloc. ancestry + Fped	-44.361	11.031
58	Transloc. ancestry	-46.544	11.499
59	Sex	-47.625	11.731
60	Sex + Fped	-48.142	11.247
61	Fped	-48.517	11.817
62	Sex + Transloc. ancestry	-48.788	11.184
63	Transloc. ancestry + Fped	-50.700	11.584

Table S8. Results from the models investigating the predictors of nesting years and lifetime reproductive success. For each model (demarcated by horizontal lines), the table includes information on each model's response variable, the parameters included in the model, and the following information for each parameter: estimate, lower and upper 95% credible intervals, $\hat{R}$ (Rhat), bulk effective sample size (ESS), and tail ESS. If not indicated with *[rand. effect]*, the parameter represents a fixed effect in the model.

Response	Parameter	Estimate	lower 95% CI	upper 95% CI	Rhat	Bulk ESS	Tail ESS
Lifetime repro. success	Intercept	-0.626	-1.350	0.098	1.000	11386.159	14968.38
	Mean group size	0.889	0.650	1.126	1.000	11573.225	14181.66
	First breeding year	-0.067	-0.099	-0.035	1.000	9782.321	13087.05
	Sex (female)	-0.235	-0.500	0.025	1.000	11454.772	14695.03
	Transloc. ancestry	1.049	0.479	1.643	1.000	8735.298	11993.66
	Ancestry count	-0.262	-0.545	0.027	1.000	10738.194	14510.05
	Fped	1.194	-5.117	7.594	1.000	19806.882	15877.01
	sd(Intercept) [rand. effect]	0.988	0.823	1.167	1.001	6479.794	11966.61
Nesting years	Intercept	0.629	0.156	1.096	1.000	19715.144	15978.47
	Mean group size	0.432	0.269	0.597	1.000	19789.331	15437.69
	First breeding year	-0.043	-0.063	-0.023	1.000	12096.918	13636.40
	Sex (female)	-0.138	-0.313	0.033	1.000	16398.614	14442.29
	Transloc. ancestry	0.541	0.206	0.888	1.000	12048.770	14016.08
	Ancestry count	-0.118	-0.313	0.075	1.000	15561.535	15019.31
	Fped	3.034	-1.813	7.838	1.000	23103.598	16119.46
	sd(Intercept) [rand. effect]	0.484	0.380	0.599	1.001	7158.109	12090.06

Table S9. Results from the models investigating the predictors of mean annual reproductive success. The models were identical except for the term(s) included in the hurdle component of the model. For each model (demarcated by horizontal lines), the table includes information on the term included in the hurdle component, the parameters included in the model, and the following information for each parameter: estimate, lower and upper 95% credible intervals, $\hat{R}$ (Rhat), bulk effective sample size (ESS), and tail ESS. If not indicated with *[rand. effect]*, the parameter represents a fixed effect in the model. The parameters associated with the hurdle component of the model are indicated with the word *hurdle*.

Hurdle term	Parameter	Estimate	lower 95% CI	upper 95% CI	Rhat	Bulk ESS	Tail ESS
Intercept only	Intercept	-0.954	-1.283	-0.630	1.001	32912.106	14643.158
	Intercept (hurdle)	-1.863	-2.819	-1.338	1.001	4282.200	4268.051
	Mean group size	0.358	0.239	0.479	1.001	34672.533	15249.859
	First breeding year	0.010	-0.002	0.022	1.000	19681.979	16786.562
	Ancestry count	-0.006	-0.129	0.118	1.000	21397.033	15661.408
	Transloc. ancestry	0.029	-0.156	0.217	1.000	23299.322	15826.622
	Fped	-1.550	-5.041	2.094	1.000	29426.718	14778.717
	Sex (female)	-0.005	-0.120	0.112	1.000	32722.286	14990.821
	sd(Intercept) [rand. effect]	0.066	0.003	0.180	1.001	4263.073	6533.786
	sd(hurdle Intercept) [rand. effect]	1.073	0.069	2.846	1.002	1750.209	3329.039
First breeding year	Intercept	-0.957	-1.283	-0.627	1.000	28267.924	15523.403
	Intercept (hurdle)	-2.204	-4.766	-1.045	1.002	1913.990	1987.925
	Mean group size	0.359	0.239	0.481	1.000	27426.652	14596.021
	First breeding year	0.010	-0.002	0.022	1.000	18288.742	15672.492
	Ancestry count	-0.006	-0.128	0.119	1.000	19589.392	15284.983
	Transloc. ancestry	0.028	-0.160	0.223	1.000	20955.647	15538.580
	Fped	-1.579	-5.003	2.059	1.000	25839.898	15771.271
	Sex (female)	-0.004	-0.119	0.110	1.000	25813.448	14921.013
	First breeding year (hurdle)	0.016	-0.050	0.123	1.001	3570.498	2816.521
	sd(Intercept) [rand. effect]	0.066	0.002	0.179	1.000	4414.032	7197.438
	sd(hurdle Intercept) [rand. effect]	1.421	0.086	4.354	1.003	1135.703	1692.189
Transloc. ancestry	Intercept	-0.956	-1.280	-0.629	1.000	28602.558	15027.543
	Intercept (hurdle)	-1.757	-2.842	-1.129	1.000	4963.159	4902.776
	Mean group size	0.359	0.238	0.479	1.000	29664.507	14285.198
	First breeding year	0.010	-0.002	0.022	1.000	18050.007	14842.204
	Ancestry count	-0.006	-0.132	0.119	1.000	18378.694	13905.818
	Transloc. ancestry	0.029	-0.161	0.222	1.000	21322.754	14955.381
	Fped	-1.559	-5.019	2.090	1.000	25541.227	14467.450
	Sex (female)	-0.004	-0.121	0.110	1.000	28296.949	14108.927
	Transloc. ancestry (hurdle)	-0.585	-2.072	0.662	1.000	11932.457	7595.859
	sd(Intercept) [rand. effect]	0.066	0.003	0.176	1.000	4241.715	6426.439
	sd(hurdle Intercept) [rand. effect]	1.220	0.103	3.310	1.001	1791.323	3113.018
Mean group size	Intercept	-0.954	-1.288	-0.632	1.000	29573.313	16205.808
	Intercept (hurdle)	0.269	-1.980	2.643	1.000	25462.617	13172.946
	Mean group size	0.358	0.239	0.480	1.000	27565.368	14273.991
	First breeding year	0.010	-0.002	0.022	1.000	18389.171	15870.072
	Ancestry count	-0.006	-0.131	0.118	1.000	19612.141	15196.918
	Transloc. ancestry	0.030	-0.158	0.224	1.000	20618.685	14790.221
	Fped	-1.547	-4.997	2.138	1.000	25585.575	15577.095
	Sex (female)	-0.004	-0.119	0.112	1.000	29559.624	14991.505
	Mean group size (hurdle)	-0.931	-2.043	-0.010	1.000	12590.513	8318.996
	sd(Intercept) [rand. effect]	0.066	0.003	0.178	1.001	4355.493	8569.826
	sd(hurdle Intercept) [rand. effect]	1.189	0.085	3.191	1.002	1573.511	2566.605
Fped	Intercept	-0.957	-1.285	-0.631	1.000	25600.221	15059.059
	Intercept (hurdle)	-1.762	-2.704	-1.269	1.001	3733.550	3130.270
	Mean group size	0.359	0.236	0.482	1.000	24859.096	15210.079
	First breeding year	0.010	-0.002	0.022	1.000	15575.221	15337.067
	Ancestry count	-0.005	-0.131	0.118	1.000	16691.600	13927.612
	Transloc. ancestry	0.030	-0.157	0.223	1.000	17975.443	15310.598
	Fped	-1.553	-5.011	2.135	1.000	23506.544	15051.938
	Sex (female)	-0.004	-0.118	0.110	1.000	25438.609	15004.920
	Fped (hurdle)	-39.422	-109.372	5.981	1.000	15464.595	9350.160
	sd(Intercept) [rand. effect]	0.067	0.003	0.179	1.001	4092.050	6747.770
	sd(hurdle Intercept) [rand. effect]	0.915	0.045	2.760	1.002	1685.273	2689.117

Sex	Intercept	-0.956	-1.278	-0.630	1.000	31275.870	15582.011
	Intercept (hurdle)	-2.216	-3.573	-1.467	1.001	3271.089	3303.041
	Mean group size	0.358	0.239	0.480	1.000	31161.014	14754.943
	First breeding year	0.010	-0.002	0.022	1.000	18597.858	16502.461
	Ancestry count	-0.007	-0.130	0.116	1.000	20666.906	15067.926
	Transloc. ancestry	0.028	-0.159	0.218	1.000	22381.239	15690.894
	Fped	-1.561	-5.004	2.093	1.000	28586.496	15613.394
	Sex (female)	-0.004	-0.118	0.111	1.000	30412.986	15739.462
	Sex; female (hurdle)	0.564	-0.265	1.527	1.001	13610.708	7607.444
	sd(Intercept) [rand. effect]	0.066	0.003	0.179	1.001	4324.380	6867.633
	sd(hurdle Intercept) [rand. effect]	1.175	0.072	3.242	1.003	1371.494	2530.437
Ancestry count	Intercept	-0.957	-1.288	-0.632	1.000	37951.468	13490.560
	Intercept (hurdle)	-2.002	-4.271	-0.762	1.001	3379.478	3957.850
	Mean group size	0.358	0.238	0.481	1.000	48738.973	13377.625
	First breeding year	0.010	-0.002	0.022	1.000	28940.412	16120.546
	Ancestry count	-0.006	-0.129	0.118	1.000	30747.590	16222.772
	Transloc. ancestry	0.029	-0.162	0.222	1.000	31689.152	14899.824
	Fped	-1.568	-4.978	2.038	1.000	38706.115	14339.508
	Sex (female)	-0.004	-0.117	0.110	1.000	44402.846	14760.972
	Ancestry count (hurdle)	0.043	-0.700	1.063	1.000	6065.102	5721.278
	sd(Intercept) [rand. effect]	0.067	0.002	0.182	1.001	5285.489	8935.991
	sd(hurdle Intercept) [rand. effect]	1.268	0.075	3.696	1.002	1857.457	3026.285

Table S10. Model structures ranked using Akaike Information Criterion corrected for sample size (AICc), relative AICc (DeltaAICc), Akaike weight (weight), number of parameters (npar), and Deviance reported for the full set of models used to examine survival differences for translocated birds vs. birds with 0% translocation ancestry. The highest supported model was used to generate apparent survival probabilities; this model included additive effects of sex and whether the individual was a directly translocated bird (translocated) on survival (Phi) and a constant detection probability (p).

model	AICc	DeltaAICc	weight	npar	Deviance
Phi(~sex + translocated)p(~1)	2106.30	0.00	5.50e-01	4	802.03
Phi(~sex * translocated)p(~1)	2107.26	0.96	3.40e-01	5	800.99
Phi(~sex)p(~1)	2110.97	4.67	5.33e-02	3	808.71
Phi(~translocated)p(~1)	2111.01	4.71	5.22e-02	3	808.75
Phi(~1)p(~1)	2115.76	9.46	4.86e-03	2	815.51
Phi(~sex + translocated)p(~time)	2132.57	26.27	1.10e-06	31	773.13
Phi(~sex * translocated)p(~time)	2133.63	27.33	6.00e-07	32	772.11
Phi(~sex + translocated + time)p(~1)	2133.75	27.46	6.00e-07	31	774.32
Phi(~sex + time)p(~1)	2136.86	30.56	1.00e-07	30	779.50
Phi(~sex)p(~time)	2137.06	30.76	1.00e-07	30	779.70
Phi(~translocated)p(~time)	2137.28	30.99	1.00e-07	30	779.92
Phi(~time + translocated)p(~1)	2139.03	32.73	0.00e+00	30	781.66
Phi(~1)p(~time)	2141.85	35.55	0.00e+00	29	786.56
Phi(~time)p(~1)	2142.50	36.20	0.00e+00	29	787.21
Phi(~sex * time)p(~1)	2153.12	46.82	0.00e+00	57	738.83
Phi(~sex + translocated + time)p(~time)	2161.13	54.83	0.00e+00	58	744.68
Phi(~sex + time)p(~time)	2163.84	57.54	0.00e+00	57	749.55
Phi(~time)p(~time)	2169.45	63.15	0.00e+00	56	757.30
Phi(~time * translocated)p(~1)	2170.44	64.14	0.00e+00	57	756.14
Phi(~sex * time)p(~time)	2181.75	75.45	0.00e+00	84	708.60
Phi(~time + translocated)p(~time)	2183.65	77.35	0.00e+00	57	769.36
Phi(~time * translocated)p(~time)	2217.49	111.19	0.00e+00	84	744.34
Phi(~sex * translocated * time)p(~1)	2229.29	122.99	0.00e+00	113	690.69
Phi(~sex * translocated * time)p(~time)	2262.07	155.77	0.00e+00	140	660.33

Table S11. Parameter estimates (on the logit scale) from the best supported of the models reported in Table S10: Phi($\sim$sex + translocated)p($\sim$1). These models were built to explore whether translocated birds showed differences in survival relative to birds with no translocation ancestry.

Parameter type	Parameter	Estimate	Standard error	lower 95% CI	upper 95% CI
Phi	Intercept	0.89	0.083	0.730	1.10
Phi	sex (male)	0.29	0.110	0.071	0.51
Phi	translocated	0.49	0.200	0.100	0.88
p	Intercept	3.90	0.230	3.400	4.40

Table S12. Model structures ranked using Akaike Information Criterion corrected for sample size (AICc), relative AICc (DeltaAICc), Akaike weight (weight), number of parameters (npar), and Deviance reported for the full set of models used to examine the relationship between translocation ancestry and survival among locally hatched birds. The highest supported model was used to generate apparent survival probabilities; this model included additive effects of sex and translocation ancestry on survival (phi) and a constant detection probability (p).

model	AICc	DeltaAICc	weight	npar	Deviance
Phi(~sex + transloc)p(~1)	2878.65	0.00	6.70e-01	4	2870.64
Phi(~sex * transloc)p(~1)	2880.66	2.01	2.46e-01	5	2870.63
Phi(~sex)p(~1)	2884.56	5.91	3.49e-02	3	984.59
Phi(~sex + time)p(~1)	2884.66	6.00	3.33e-02	30	929.86
Phi(~sex + transloc + time)p(~1)	2886.54	7.88	1.30e-02	31	2823.64
Phi(~transloc)p(~1)	2889.91	11.25	2.41e-03	3	2883.90
Phi(~time)p(~1)	2896.40	17.75	9.38e-05	29	943.66
Phi(~1)p(~1)	2896.54	17.88	8.76e-05	2	998.58
Phi(~sex + transloc)p(~time)	2897.01	18.36	6.92e-05	31	2834.12
Phi(~time + transloc)p(~1)	2898.20	19.55	3.82e-05	30	2837.36
Phi(~sex * transloc)p(~time)	2899.07	20.41	2.47e-05	32	2834.12
Phi(~sex)p(~time)	2902.78	24.13	3.90e-06	30	947.99
Phi(~sex + time)p(~time)	2902.91	24.26	3.60e-06	57	891.95
Phi(~transloc)p(~time)	2908.18	29.53	3.00e-07	30	2847.35
Phi(~sex * time)p(~1)	2913.90	35.25	0.00e+00	57	902.93
Phi(~1)p(~time)	2914.86	36.20	0.00e+00	29	962.12
Phi(~time + transloc)p(~time)	2916.26	37.61	0.00e+00	57	2799.25
Phi(~sex + transloc + time)p(~time)	2923.38	44.73	0.00e+00	58	2804.27
Phi(~time)p(~time)	2933.17	54.52	0.00e+00	56	924.31
Phi(~time * transloc)p(~1)	2941.02	62.37	0.00e+00	57	2824.01
Phi(~sex * time)p(~time)	2942.18	63.53	0.00e+00	84	873.64
Phi(~time * transloc)p(~time)	2960.86	82.20	0.00e+00	84	2786.27
Phi(~sex * transloc * time)p(~1)	2998.72	120.07	0.00e+00	113	2760.68
Phi(~sex * transloc * time)p(~time)	3030.32	151.67	0.00e+00	140	2731.64

Table S13. Parameter estimates (on the logit scale) from the best supported of the models reported in Table S12: Phi(∼sex + transloc)p(∼1). These models were built to explore whether survival varied based on an individual's proportion of translocation ancestry.

Parameter type	Parameter	Estimate	Standard error	lower 95% CI	upper 95% CI
Phi	Intercept	0.85	0.083	0.69	1.00
Phi	sex (male)	0.36	0.099	0.17	0.56
Phi	transloc	0.47	0.170	0.14	0.80
p	Intercept	3.30	0.160	3.00	3.70

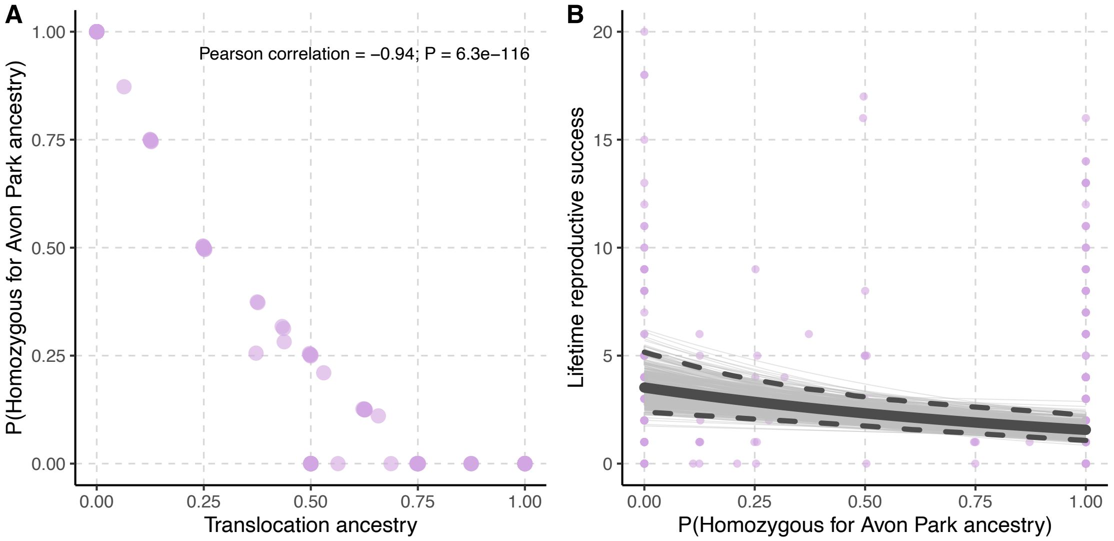

Figure S9. As discussed in the main text, a potential explanation for the positive relationship between translocation ancestry and lifetime reproductive is that more translocation ancestry is associated with the masking of deleterious recessive mutations at high frequency in the Avon Park population. If so, we would expect that the extent of a bird's translocation ancestry is closely connected to its probability of homozygosity for Avon Park ancestry [P(Homozygous for Avon Park ancestry)], and P(Homozygous for Avon Park ancestry) would show a negative relationship with lifetime reproductive success. In panel A, we verify that translocation ancestry shows a strong, negative correlation with P(Homozygous for Avon Park ancestry). (B) The model reported in Table S14 supports a negative relationship between P(Homozygous for Avon Park ancestry) and lifetime reproductive success. The bold, solid line shows the mean predicted relationship, the dashed lines represent the 95% credible interval, and the lighter gray lines represented the predicted relationship based on 500 draws from the model's posterior distribution. The purple points represent the raw data.

Table S14. Parameter estimates for a model built to explore the relationship between an individual's probability for homozygous Avon Park ancestry [P(Homozygous for Avon Park ancestry)] and lifetime reproductive success and is intended as a complement to the main text's models, which considered translocation ancestry instead of P(Homozygous for Avon Park ancestry). This relationship is visualized in Fig. S9B.

Response	Parameter	Estimate	lower 95% CI	upper 95% CI	Rhat	Bulk ESS	Tail ESS
lifetime repro. success	Intercept	0.335	-0.497	1.165	1	11001.528	14131.21
	Fped	1.968	-4.341	8.388	1	19780.832	16834.20
	Mean group size	0.926	0.693	1.161	1	11912.823	14811.62
	First breeding year	-0.060	-0.090	-0.030	1	10285.530	12938.93
	Sex (female)	-0.207	-0.469	0.050	1	11371.853	14360.87
	P(Homozygous for Avon Park ancestry)	-0.830	-1.327	-0.352	1	9192.515	13169.42
	Ancestry count	-0.476	-0.758	-0.201	1	10702.632	13252.75
	sd(Intercept) [rand. effect]	0.975	0.811	1.159	1	6534.207	11273.03

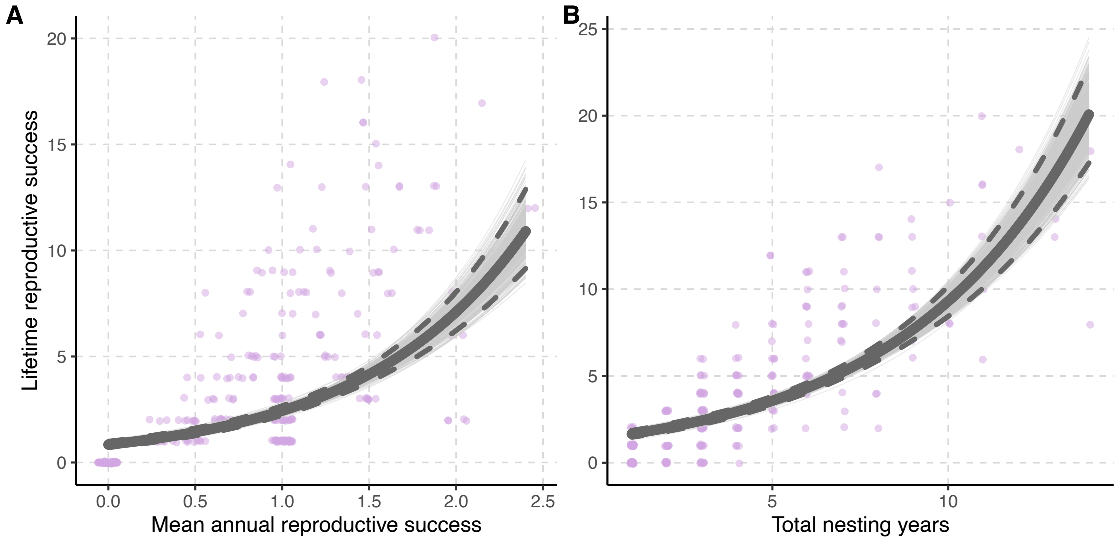

Figure S10. Estimated relationships between lifetime reproductive success and both mean annual reproductive success (A) and total nesting years (B). For each parameter estimate, the bold, solid line shows the mean predicted relationship, the dashed lines represent the 95% credible interval, and the lighter gray lines represented the predicted relationship based on 500 draws from the model's posterior distribution. The purple points represent the raw data and are visualized with a slight jitter so that overlapping points can be more clearly viewed. The parameter estimates for this model (as well as other information) are included in Table S15.

Table S15. Results for the model built to verify that both mean annual reproductive success and total nesting years show positive relationships with lifetime reproductive success. Confirming these relationships helped strengthen our ability to explore how the relationship between translocation ancestry and lifetime reproductive success arose (see the main text for further discussion). For each parameter included in the model, the table includes its estimate, lower and upper 95% credible interval, Rhat, and bulk and tail effective sample sizes (ESS). This model is visualized in Fig. S10.

Response	Parameter	Estimate	lower 95% CI	upper 95% CI	Rhat	Bulk ESS	Tail ESS
Lifetime repro. success	Intercept	-0.747	-0.942	-0.556	1	23266.85	15855.99
	Mean annual reproductive success	1.066	0.943	1.190	1	28836.26	15080.54
	Total nesting years	0.191	0.173	0.208	1	29359.72	16052.53
	sd(Intercept) [rand. effect]	0.036	0.002	0.100	1	13697.68	11080.77

Table S16. Results for a model that was identical to lifetime reproductive success model reported in the main text but was re-fit with a dataset reduced down to individuals with complete grandparent information. We estimated this model to explore whether the inclusion of individuals with insufficient ancestor information to detect inbreeding influenced our ability to detect a relationship between pedigree inbreeding and lifetime reproductive success. For each model parameter, the table includes its estimate, lower and upper 95% credible intervals, Rhat, and bulk and tail effective sample sizes (ESS)

Response	Parameter	Estimate	lower 95% CI	upper 95% CI	Rhat	Bulk ESS	Tail ESS
Lifetime repro. success	Intercept	0.094	-1.909	2.043	1.000	7662.038	11708.08
	Mean group size	0.925	0.400	1.469	1.000	7659.714	10949.55
	First breeding year	-0.085	-0.185	0.014	1.000	6799.370	10554.60
	Sex (female)	-0.527	-1.076	-0.001	1.001	7268.172	10499.50
	Transloc. ancestry	0.268	-3.051	3.478	1.000	7327.744	10411.59
	Ancestry count	0.043	-0.922	1.033	1.000	7328.018	10350.37
	Fped	2.464	-5.094	9.841	1.000	12041.594	14176.66
	sd(Intercept) [rand. effect]	1.128	0.811	1.518	1.000	6149.568	10322.45

Reproductive success and genetic contributions

To aid in our interpretation of the variation in expected genetic contributions of the translocated birds, we explored whether the lifetime reproductive success of translocated birds corresponded to differences in lifetime reproductive success with the hypothesis that those with higher lifetime reproductive success tended to have larger genetic contributions. As a preliminary approach to address this question, we focused on the 34 birds that established in the population after translocation and were no longer alive (based on the census data) in the final monitoring year and examined the relationship between lifetime reproductive success (total number of fledglings produced by a bird over its lifetime) and expected genetic contribution in the final monitoring year (2022). We focused on the final monitoring year because all translocation events had occurred at least several years before this year (and thus we could consider all translocation cohorts and nearly all translocated individuals) and this seemed like a natural time point to evaluate the state of the population. We fit a mixed effects model with the `R` package `glmmTMB` (15). The model included 2022 genetic contribution as the response and lifetime reproductive success as a fixed effect predictor. We included a random intercept term for cohort identity. This represents a simple approach to account for the non-independent nature of how birds were translocated into Avon Park but does not allow us to explicitly evaluate other aspects of the translocations that could influence genetic contributions such as differences in the timing of translocations. We fit the model as an ordered beta regression (the *ordbeta* family in `glmmTMB`; (16)). We chose this modeling approach because genetic contributions can span the closed interval [0, 1], which the ordered beta model accommodates. This model provided clear support for a positive relationship between an individual's lifetime reproductive success and its magnitude of genetic contribution in the final monitoring year (parameter estimate for lifetime reproductive success fixed effect: 0.104; z value = 4.336; P = 1.45e-05) suggesting that variation in reproductive success represents a partial (and proximate) explanation for the variation in genetic contributions among translocated birds.

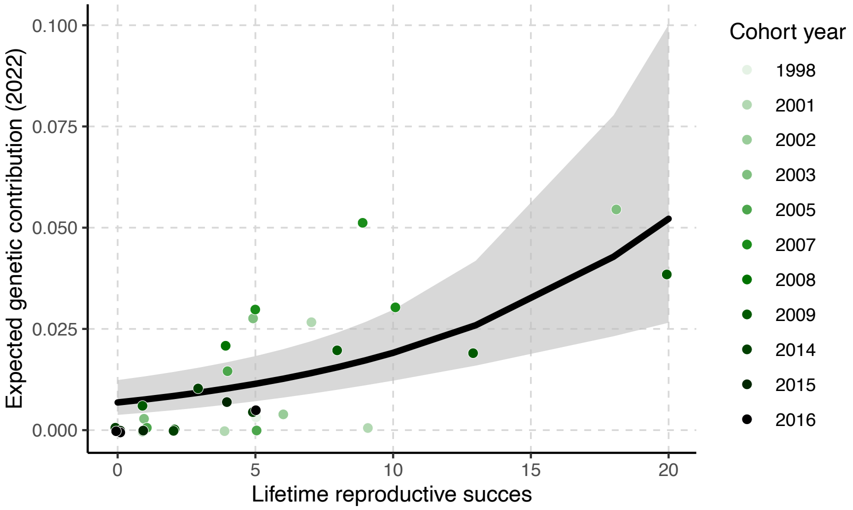

Figure S11. The estimated relationship between lifetime reproductive success of translocated birds that died prior to the last monitoring year and their expected genetic contributions in the final monitoring year (2022) based on a mixed effects ordered beta regression with a random effect of cohort. The gray band represents the 95% confidence interval. The points represent the raw data colored based on cohort (coloration is darker for more recent cohorts), and their positions are slightly jittered so that overlapping points can be seen.

References

[1] RM Cormack, Estimates of survival from the sighting of marked animals. *Biometrika* **51**, 429–438 (1964).

[2] GM Jolly, Explicit estimates from capture-recapture data with both death and immigration-stochastic model. *Biometrika* **52**, 225–248 (1965).

[3] GAF Seber, A note on the multiple-recapture census. *Biometrika* **52**, 249–260 (1965).

[4] GC White, KP Burnham, Program MARK: survival estimation from populations of marked animals. *Bird Study* **46**, S120–S139 (1999).

[5] JL Laake, RMark: An R interface for analysis of capture-recapture data with MARK (2013).

[6] KP Burnham, DR Anderson, *Model selection and multimodel inference: a practical information-theoretic approach.* (Springer-Verlag, New York), 2nd edition, (2002).

[7] D Fletcher, Estimating overdispersion when fitting a generalized linear model to sparse data. *Biometrika* pp. 230–237 (2012).

[8] A Gelman, D Lee, J Guo, Stan: A Probabilistic Programming Language for Bayesian Inference and Optimization. *J. Educ. Behav. Stat.* **40**, 530–543 (2015).

[9] PC Bürkner, brms: An R Package for Bayesian Multilevel Models Using Stan. *J. Stat. Softw.* **80**, 1–28 (2017).

[10] JR Walters, Application of Ecological Principles to the Management of Endangered Species: The Case of the Red-Cockaded Woodpecker. *Annu. Rev. Ecol. Syst.* **22**, 505–523 (1991).

[11] A Vehtari, A Gelman, J Gabry, Practical Bayesian model evaluation using leave-one-out cross-validation and WAIC. *Stat. Comput.* **27**, 1413–1432 (2017).

[12] A Vehtari, A Gelman, J Gabry, loo: Efficient leave-one-out cross-validation and WAIC for Bayesian models (2024).

[13] A Vehtari, Cross-validation FAQ (2024).

[14] M Kay, tidybayes: Tidy Data and Geoms for Bayesian Models (2024).

[15] ME Brooks, et al., glmmTMB balances speed and flexibility among packages for zero-inflated generalized linear mixed modeling. *The R J.* **9**, 378–400 (2017).

[16] R Kubinec, Ordered Beta Regression: A Parsimonious, Well-Fitting Model for Continuous Data with Lower and Upper Bounds. *Polit. Analysis* **31**, 519–536 (2022).
